# Assessing the Potential Distribution of *Eriocheir sinensis* Under Climate Change: Regional Contrasts and Spatial Mismatches in Terrestrial and Nearshore Suitability

**DOI:** 10.1002/ece3.74351

**Published:** 2026-10-16

**Authors:** He Gong, Xiaolong Yang, Gorka Bidegain, Yihang Wang, Yuqing Jiang, Tao Han, Jianyu Dong, Xiumei Zhang

**Affiliations:** ^1^ Fishery College Zhejiang Ocean University Zhoushan China; ^2^ Department of Applied Mathematics, Faculty of Engineering Gipukkoq University of the Basque Country (UPV/EHU) Leioa Spain; ^3^ Research Center for Experimental Marine Biology and Biotechnology, Plentzia Marine Station University of the Basque Country (PiE‐UPV/EHU) Plentzia Spain; ^4^ Fisheries College Guangdong Ocean University Zhanjiang China

**Keywords:** biological invasion, climate change, *Eriocheir sinensis*, invasive species, species distribution model environmental‐space dynamics

## Abstract

The Chinese mitten crab ( *Eriocheir sinensis* ), native to China and the Korean Peninsula, has established invasive populations with substantial ecological and economic impacts in Europe and North America. Anticipating changes in its potential distribution is important for invasion‐risk screening and estuarine management. We developed a global species distribution model (G‐SDM) and regional models for the native range, Europe, and North America, each comprising terrestrial and nearshore brackish‐water components. Future suitability was projected under SSP126, SSP245, and SSP585. Terrestrial PCA‐env analyses assessed changes in projected suitable environmental space, whereas MESS and MoD diagnosed environmental novelty and extrapolation uncertainty. Terrestrial suitability was mainly associated with annual mean temperature, annual precipitation, and distance to the coastline, whereas nearshore suitability was mainly associated with water depth, chlorophyll concentration, and sea‐surface temperature, although the ranking of predictor importance differed among regions. Predicted‐suitable environments in Europe and North America extended to lower annual and wettest‐quarter temperatures than those in the native range. Under future scenarios, projected suitability was generally redistributed towards higher latitudes, particularly under SSP585. Terrestrial suitability showed more pronounced spatial redistribution than nearshore suitability in several regions, and the two components were not always geographically aligned. Terrestrial PCA‐env analyses indicated greater retention of suitable environmental space in Europe but increasing unfilling in the native range and North America. MESS analysis further identified environmental novelty and extrapolation uncertainty in some future suitable areas. These findings provide a scientific basis for early prevention, targeted monitoring, field validation, and estuarine management.

## Graphical abstract

We developed global and regional species distribution models to assess current and future terrestrial freshwater and nearshore brackish‐water suitability for *Eriocheir sinensis* across its native range, Europe, and North America. Future projections indicated poleward redistribution, region‐specific reorganization of suitable environmental space, and incomplete spatial correspondence between terrestrial and nearshore suitability, with greater extrapolation uncertainty in some future areas. These results provide a basis for targeted monitoring, field validation, and estuarine management under climate change.

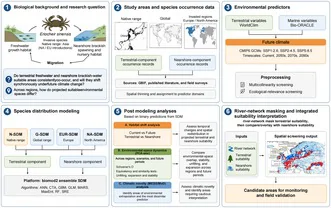

## 1 Introduction

Biological invasions are increasingly recognized as one of the most critical ecological challenges in an era of globalization (Pyšek et al. 2020). Invasive species threaten regional ecological security and stability through various mechanisms, including interspecific competition (Zhang et al. 2019b), predation (Doherty et al. 2016), habitat alteration (Byers et al. 2010), damage to infrastructure, and pathogen transmission (Dittel and Epifanio 2009). With the rapid changes in global climate, the geographical distributions and niche boundaries of both terrestrial and marine species are being reshaped at an unprecedented scale and pace (Hattab et al. 2014; Rubenstein et al. 2023). By modifying key environmental factors such as temperature and precipitation, climate change directly influences species' survival, reproduction, and dispersal (Briscoe et al. 2023; Kearney and Porter 2009). These alterations may potentially create new suitable habitats for invasive species or intensify their adverse effects in already invaded regions (Walther et al. 2009). Although the responses of invasive species to future stressors remain uncertain, their continued expansion is expected to have profound consequences for global biodiversity and ecosystem function (Guy‐Haim et al. 2018; Pyšek et al. 2020). Given the widespread occurrence and complexity of biological invasions, predicting their potential distributions and future dynamics is important for prioritizing monitoring and management (Barbet‐Massin et al. 2012), thereby contributing to marine biodiversity conservation and the objectives of UN Sustainable Development Goal 14.

Niche conservatism has long provided an important theoretical basis for predicting the potential spread and future distributions of invasive species (Wiens et al. 2010). It assumes that species retain broadly similar environmental associations across space and time, providing a basis for transferring models calibrated in the native range to non‐native regions (Liu, Wolter, et al. 2020). However, increasing evidence shows that invasive species do not always occupy the same environmental space in their native and invaded ranges (Guisan et al. 2014; Sherpa et al. 2019). Such differences do not necessarily indicate niche evolution or local adaptation, but may also arise from differences in regional environmental availability, range filling, invasion history, environmental filtering, and the geographic extent of model calibration (Atwater et al. 2018; Chevalier et al. 2022; Guisan et al. 2014). In particular, when environmental gradients and occurrence coverage differ among regions, regional models may characterize different portions of a species' realized environmental space, resulting in different environmental associations and suitability responses (Chevalier et al. 2022; Liu et al. 2022). Models calibrated solely with native‐range data may therefore provide an incomplete representation of environmental associations in different invaded regions (Liu, Wolter, et al. 2020; Liu, Wang, et al. 2020). Accordingly, independently calibrating models for the native and invaded ranges under a common environmental‐variable framework can facilitate comparisons of regional environmental associations and their projected responses to future climate change, while distinguishing modeled regional environmental differences from their potential evolutionary causes.

The Chinese mitten crab, *Eriocheir sinensis* (H. Milne Edwards, 1853), is native to coastal regions from China to the Korean Peninsula, and is a species of significant commercial importance in aquaculture, particularly in China (Liu et al. 2023). Since the early 20th century, human activities such as transoceanic ballast water discharge and the live animal trade have facilitated the introduction and establishment of this crab species in Europe, followed by its spread to North America in the 1990s, rendering it a notable aquatic invader (Herborg et al. 2005). The invasion history of *E. sinensis* differs markedly between these two continents. The first established population was recorded in Germany in 1912, followed by a gradual spread throughout major European river systems in short time, where it formed stable, self‐sustaining populations (Herborg et al. 2005). In contrast, its invasion of America is more recent, with initial records dating to the 1990s on the Pacific Coast. Despite its subsequent spread to various U.S. coastal waters and estuaries over the past three decades, evidence indicates that these populations have not yet reached the stable, high‐density levels observed in Europe (Rilov et al. 2024).

As a catadromous species, *E. sinensis* spends juvenile and growth phases in freshwater habitats before migrating to marine environments for reproduction. This distinctive “river‐to‐sea” life history makes its survival and reproductive success highly dependent on uninterrupted hydrological connectivity and specific salinity regimes that are synchronized with its phenological stages (Shen et al. 2022; Zhang et al. 2023). This life history creates a spatial separation between freshwater environments used primarily for juvenile growth and estuarine or nearshore brackish‐water environments associated with reproduction and early development. This separation implies that future climate change could impact different life stages unevenly, leading to complex changes in its overall habitat suitability (Anger 1991; Thirukanthan et al. 2023). For instance, variations in the salinity of marine conditions could directly limit reproductive success, while alterations in air temperature and freshwater hydrology are likely to have a greater impact on the growth and distribution of adults (Anger 1991). Given the classification of *E. sinensis* as a high‐concern invasive species, a systematic investigation and prediction into its differential responses to future climate change is essential.

To assess how future environmental change may alter the potential distribution of *Eriocheir sinensis* , we developed a global species distribution model (G‐SDM) using current occurrence records of *Eriocheir sinensis* from its native range and other non‐native regions, and independently calibrated three regional models using current occurrence records from the native range (N‐SDM), Europe (EUR‐SDM), and North America (NA‐SDM), respectively. The global model (G‐SDM) was used to characterize broad‐scale distributional patterns, whereas three independently calibrated regional models for the native range (N‐SDM), Europe (EUR‐SDM), and North America (NA‐SDM) were used to compare projected responses among regions. Each model comprised a terrestrial component and a nearshore brackish‐water component. Using environmental projections derived from CMIP6 global climate models, we further projected future changes in terrestrial suitability and nearshore brackish‐water environmental suitability, which may be relevant to the completion of the catadromous life cycle of *E. sinensis* . The nearshore projections were interpreted as potentially suitable environments rather than confirmed spawning or nursery habitats. We further compared regional variation in predictor importance and quantified the overlap, breadth, and temporal dynamics of predicted suitable environmental space. Accordingly, this study aimed to: (1) project future changes in terrestrial and nearshore brackish‐water environmental suitability across the native and invaded ranges; (2) compare the environmental predictors and suitability responses identified by the regional SDMs; and (3) quantify regional differences in the overlap, breadth, and temporal dynamics of predicted suitable environmental space.

## 2 Materials and Methods

The overall methodological workflow of this study is shown in Figure 1.

**FIGURE 1:**
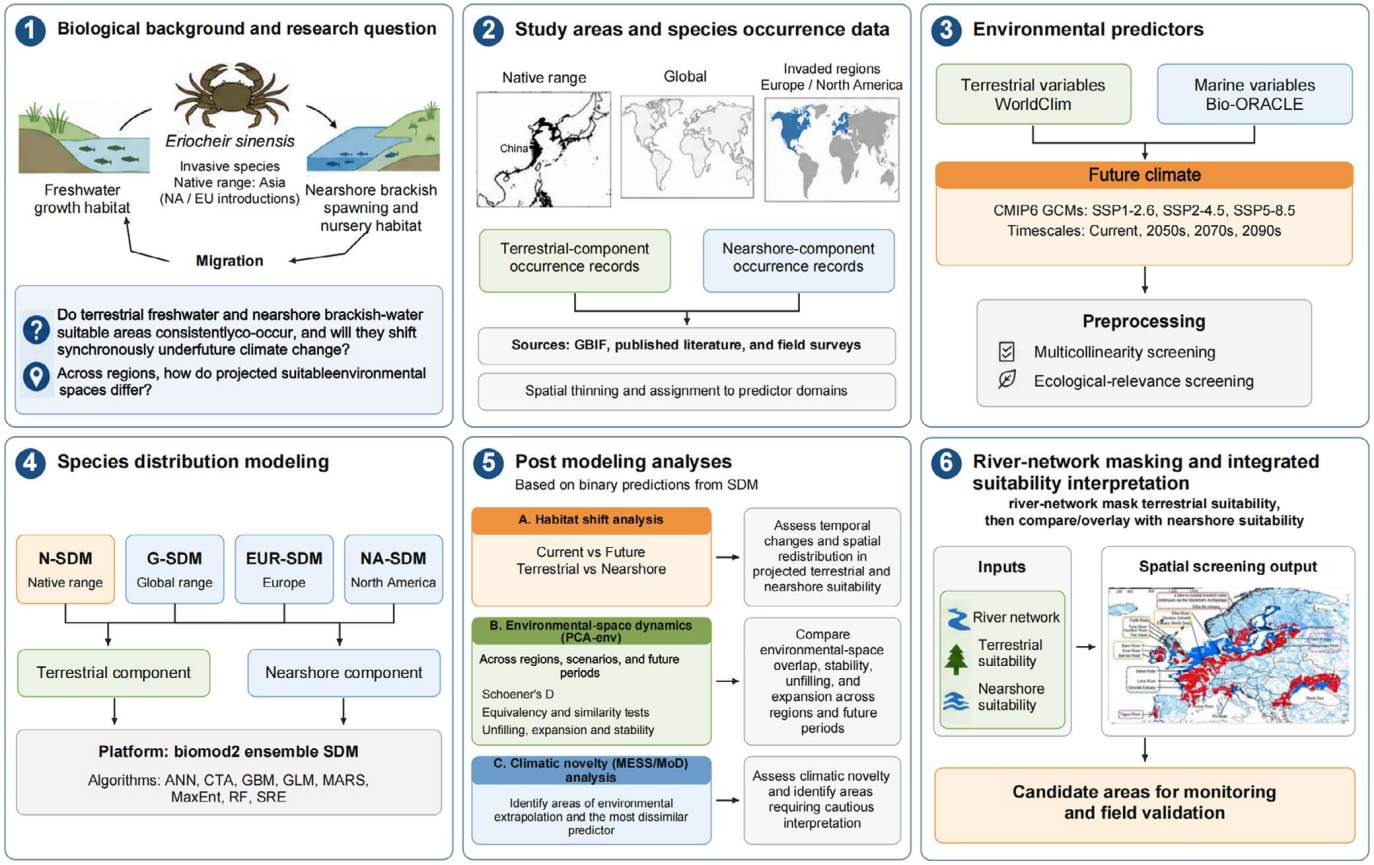
Schematic diagram of the methodological framework.

### 2.1 Modeling Domains and Species Occurrence Data

To comprehensively evaluate the potential distribution of *E. sinensis* and regional variation in its projected responses to climate change, we considered four geographic modeling domains: the Asia–Pacific native range, Europe, North America, and the global extent. The global model (G‐SDM) was used to characterize broad‐scale patterns of potential environmental suitability and projected distributional shifts worldwide. Three independently calibrated regional models—the native‐range model (N‐SDM), European model (EUR‐SDM), and North American model (NA‐SDM)—were developed using the same set of environmental predictors to facilitate direct comparisons of projected changes in habitat suitability among the native and invaded ranges (Figure 2). Each geographic model comprised a terrestrial component and a nearshore component. The terrestrial component estimated environmental suitability relevant to the freshwater‐associated phase of the species' life cycle, whereas the nearshore component estimated environmental suitability in nearshore brackish‐water environments potentially relevant to reproduction and early development (Anger 1991; Long et al. 2018).

**FIGURE 2:**
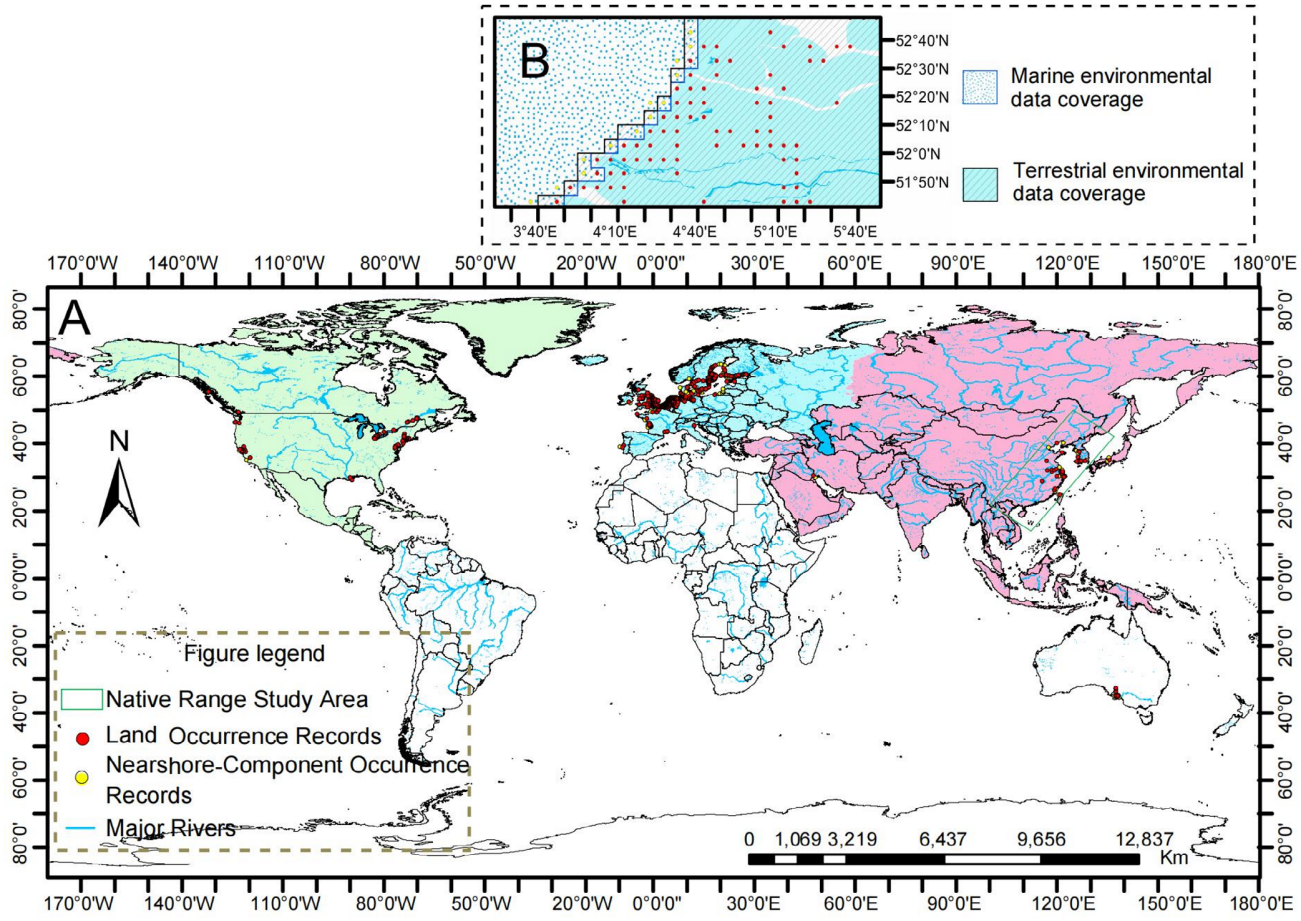
Study area; (A) Global extent, with the colored continents indicating the primary study regions; (B) Example illustrating the spatial coverage and overlap of terrestrial and marine environmental datasets in coastal areas.

Occurrence records of *E. sinensis* were obtained from the Global Biodiversity Information Facility (https://www.gbif.org; https://doi.org/10.15468/dl.42zs6y) (GBIF 2025), published literature (Zhang, Capinha, et al. 2019), and field investigation. A total of 4408 occurrence records were compiled. To reduce spatial sampling bias and spatial autocorrelation arising from record redundancy and uneven sampling density among regions, occurrence data were spatially thinned using ENMTools, retaining no more than one record per 10 × 10 km grid cell (Warren et al. 2010). The thinned records were then intersected separately with the terrestrial and nearshore environmental predictor layers. Records falling within the spatial coverage of the terrestrial predictors were assigned to the terrestrial component, whereas those falling within the coverage of the nearshore marine predictors were assigned to the nearshore component. Because the two predictor domains overlapped in some coastal areas, records located within these overlapping areas could be included in both modeling components (Figure 2B). Records assigned to the nearshore component were not direct observations of spawning or nursery activity; accordingly, outputs from this component were interpreted as predicted suitable nearshore brackish‐water environments rather than confirmed reproductive habitats. Following spatial thinning and intersection with the corresponding predictor layers, the N‐SDM retained 56 records for the terrestrial component and 31 records for the nearshore component. The G‐SDM retained 685 and 271 records for the terrestrial and nearshore components, respectively. The EUR‐SDM retained 472 terrestrial‐component records and 216 nearshore‐component records, whereas the NA‐SDM retained 67 and 28 records, respectively.

### 2.2 Acquisition and Screening of Environmental Variables

Current and future environmental predictors were obtained from WorldClim v2.1 (https://www.worldclim.org; Fick and Hijmans 2017) and Bio‐ORACLE v3.0 (https://www.bio‐oracle.org; Assis et al. 2024) for the terrestrial and nearshore components, respectively. The WorldClim dataset comprised 19 bioclimatic variables, with the current baseline representing 1970–2000. Future terrestrial projections were based on the CMCC‐ESM2 model from CMIP6 and were obtained for three periods: 2041–2060 (hereafter the 2050s), 2061–2080 (the 2070s), and 2081–2100 (the 2090s) (Fick and Hijmans 2017). Bio‐ORACLE v3.0 provides marine environmental predictors as decadal climatologies from 2000 to 2100, with future SSP projections derived from a multi‐model ensemble of CMIP6 Earth System Models (Assis et al. 2024). To harmonize the temporal resolution of the marine predictors with the approximately 20‐year periods used for the WorldClim projections, adjacent Bio‐ORACLE decadal layers were combined according to the statistical definition of each predictor for the 2050s, 2070s, and 2090s. For variables represented by decadal means, values from two consecutive decades were averaged (Serandour et al. 2024). For maximum sea‐surface temperature, the higher value of the two decadal maxima was retained to represent the maximum over the combined period. Future projections were evaluated under three Shared Socioeconomic Pathway scenarios: SSP1‐2.6, SSP2‐4.5, and SSP5‐8.5 (hereinafter referred to as: SSP126/245/585) (Riahi et al. 2017). Although the terrestrial and marine datasets differed in their current baseline periods and future temporal windows, they were used in separate terrestrial and nearshore sub‐models; therefore, predictors representing different time periods were not combined as contemporaneous covariates within the same fitted model. Distance to the nearest coastline was included as an additional terrestrial predictor (Wessel and Smith 1996), and all initial environmental variables are listed in Table S1. Because the modeling extents differed among geographic models, environmental predictors were standardized to a spatial resolution of 5 arc‐min for the G‐SDM and 2.5 arc‐min for the N‐SDM, EUR‐SDM, and NA‐SDM using bilinear interpolation.

To reduce multicollinearity among predictors, Pearson's correlation coefficients were calculated from environmental raster cells within 100‐km buffers around the occurrence records before model fitting (Barve et al. 2011; Díaz‐Vallejo et al. 2024; Fourcade et al. 2014; Galante et al. 2018). When the absolute pairwise correlation coefficient exceeded 0.75 (|*r*| > 0.75), the variable considered more ecologically relevant to *E. sinensis* was retained (Díaz‐Vallejo et al. 2024; Herborg et al. 2005; Zhang et al. 2020). This procedure retained 12 terrestrial and 6 marine variables for the G‐SDM, and 8 terrestrial and 6 marine variables for each regional model (s Table S1, Figure S1; Table 1).

**TABLE 1:** Environmental variables included in the three region models and global model (G‐SDM).

Terrestrial variables (G‐SDM/three region‐SDM)		Marine variables (G‐SDM/three region‐SDM)	
Bio1	Annual Mean Temperature (√/√)	M‐Bio4	Water Depth (√/√)
Bio2	Diurnal Temperature Range (√/√)	M‐Bio8	pH Mean (√/√)
Bio3	Isothermality (√/√)	M‐Bio9	Phosphate Mean (×/√)
Bio5	Maximum Temperature of Warmest Month (√/×)	M‐Bio10	Salinity Mean (√/√)
Bio6	Minimum Temperature of Coldest Month (√/×)	M‐Bio12	Surface Sea Temperature Maximum (√/×)
Bio7	Temperature Annual Range (√/×)	M‐Bio13	Surface Sea Temperature Mean (√/√)
Bio8	Mean Temperature of Wettest Quarter (×/√)	M‐Bio15	Chlorophyll Mean (√/√)
Bio12	Annual Precipitation (×/√)		
Bio15	Precipitation Seasonality (√/√)		
Bio16	Precipitation of Wettest Quarter (√/×)		
Bio17	Precipitation of Driest Quarter (√/×)		
Bio18	Precipitation of Warmest Quarter (√/√)		
Bio19	Precipitation of Coldest Quarter (√/×)		
Bio20	Distance to Coastline (√/√)		

*Note:* Checkmarks (√) and crosses (×) denote the inclusion or exclusion of each variable in the respective model.

### 2.3 Modeling Procedure

For each geographic SDM (G‐SDM, N‐SDM, EUR‐SDM, and NA‐SDM), separate terrestrial and nearshore components were developed in R using the “biomod2” package (v: 4.2–3) (Thuiller et al. 2009). Eight algorithms were implemented: artificial neural networks (ANN), classification tree analysis (CTA), generalized boosting models (GBM), generalized linear models (GLM), multivariate adaptive regression splines (MARS), maximum entropy (MaxEnt), random forests (RF), and surface range envelope (SRE) (Thuiller et al. 2009). MaxEnt model complexity was tuned prior to fitting the final models (Muscarella et al. 2014). The candidate parameter combinations, selection criteria, and final settings are described in the Supporting Information (Figure S2, Method). The remaining algorithms were fitted using the default settings in biomod2. Because confirmed absence records were unavailable, pseudo‐absences were randomly sampled from raster cells with complete environmental predictor values within the predefined calibration domain of each geographic model (Wisz and Guisan 2009). No minimum‐distance buffer or additional environmental filtering was applied during pseudo‐absence selection. For each terrestrial and nearshore component of the G‐SDM, three independent pseudo‐absence datasets were generated, each containing 5000 pseudo‐absences. For each component of the N‐SDM, EUR‐SDM, and NA‐SDM, three datasets containing 1000 pseudo‐absences each were generated. Multiple pseudo‐absence datasets were used to reduce sensitivity to any single random selection of background locations (Barbet‐Massin et al. 2012; Guisan et al. 2017).

For each pseudo‐absence dataset, model calibration and internal evaluation were repeated ten times using repeated random holdout partitioning. In each repetition, 75% of the occurrence and pseudo‐absence records were used for model calibration, and the remaining 25% were used for evaluation (Acosta et al. 2016; Paterson et al. 2024). This design produced 240 individual models for each component of each geographic model (8 algorithms × 3 pseudo‐absence datasets × 10 calibration repetitions). Model performance was evaluated using the true skill statistic (TSS) and the area under the receiver operating characteristic curve (AUC) (Allouche et al. 2006; Konowalik and Nosol 2021; Rana et al. 2025). Models satisfying both predefined criteria (TSS > 0.85 and AUC > 0.85) were retained for ensemble modeling (Allouche et al. 2006; Phillips et al. 2017). Because random holdout partitioning does not adequately account for spatial dependence among species occurrence records, spatial block cross‐validation was additionally conducted for the four geographic SDMs to assess the robustness of model performance (Roberts et al. 2017; Valavi et al. 2019). Occurrence and pseudo‐absence records were partitioned into spatially separated folds, with each fold withheld in turn for validation while the remaining folds were used for model calibration. Model discrimination under spatially separated validation was evaluated using TSS and ROC‐AUC. This analysis was used solely as an additional robustness assessment and was not used for individual‐model selection, ensemble construction, threshold determination, or spatial projection.

The retained individual models were combined using the ensemble mean (EMmean), ensemble median (EMmedian), and evaluation‐score‐weighted mean (EMwmean). The discrimination performance of each ensemble model was evaluated using TSS (Konowalik and Nosol 2021; Marmion et al. 2009). The ensemble method with the highest TSS was selected for subsequent spatial projection. For cartographic visualization, continuous suitability predictions were divided into four classes using the Jenks natural‐breaks method in ArcGIS 10.4.1 (Jenks 1967). This classification was used only for map presentation. For binary analyses, the TSS‐maximizing probability threshold was determined separately for each geographic‐model component and then applied to its corresponding current and future projections (Liu, Wang, et al. 2020; Shim et al. 2023).

As a post‐processing analysis, ArcGIS v. 10.8.1 was used to spatially screen river‐associated areas within the predicted suitable regions of Europe and North America. River‐network data were obtained from HydroRIVERS (https://www.hydrosheds.org), a global river‐network product of the HydroSHEDS database (Lehner and Grill 2013). River reaches with classical river orders (ORD_CLAS) of 1–3 were retained, representing main‐stem rivers and their first two tributary levels. Tributary sub‐basins intersecting predefined 50‐km buffers around selected major rivers were first extracted and overlaid with the binary terrestrial SDM projections. Cells classified as moderately or highly suitable within the screening areas were retained, whereas coastal areas lacking corresponding predicted suitable nearshore brackish‐water environments were excluded (Figure 15). The 50‐km buffer served solely as a predefined spatial screening extent and does not represent the actual dispersal distance of *E. sinensis* . This analysis identified areas where predicted terrestrial suitability, river systems, and suitable nearshore brackish‐water environments spatially coincided, thereby highlighting potential areas containing environmental conditions relevant to different stages of the *E. sinensis* life cycle (Anger 1991; Rudnick and Resh 2005). It was not intended to represent hydrological connectivity or actual dispersal pathways.

### 2.4 Projected Environmental‐Space Dynamics of *Eriocheir sinensis* Under Future Climate Scenarios

To quantify temporal changes in the projected suitable terrestrial environmental space of *E. sinensis* , we applied the PCA‐env framework to the native range, Europe, and North America using only the terrestrial components of the regional SDMs (Broennimann et al. 2012; Di Cola et al. 2017; Li et al. 2014). We focused the PCA‐env analysis on the terrestrial component because *E. sinensis* spends most of its post‐larval life in freshwater systems (Rudnick and Resh 2005). Binary SDM projections were used to characterize model‐derived suitable environmental space, with cells coded as 1 representing predicted suitable conditions and cells coded as 0 representing predicted unsuitable conditions (Wani et al. 2022).

The same seven bioclimatic variables used for the regional SDMs—bio01, bio02, bio03, bio08, bio12, bio15, and bio18—were applied consistently across all regions and time periods. A single principal component analysis (PCA) was calibrated using equal‐sized random samples of 20,543 grid cells from the current environmental background of each region (native range, Europe, and North America), thereby establishing a common contemporary environmental reference (Broennimann et al. 2012; Di Cola et al. 2017). All variables were centered and standardized using the means and standard deviations of the current‐background samples. The resulting scaling parameters and PCA loadings were then held constant when projecting the current and all nine future scenarios, ensuring that all region–period combinations were represented within the same PC1–PC2 coordinate system. To avoid clipping projected future scores falling outside the current environmental extent, the boundaries of a common 100 × 100 PCA‐env grid were defined using the combined range of projected current and future scores, while environmental availability was characterized separately for each region–period combination (Broennimann et al. 2012; Di Cola et al. 2017). Predicted suitable cells from the binary SDMs were subsequently used to estimate model‐derived suitability‐density distributions in environmental space and to quantify current–future environmental‐space overlap and dynamics. Environmental‐space overlap was quantified using Schoener's D, which ranges from 0 (no overlap) to 1 (complete overlap) (Broennimann et al. 2012).

Environmental‐space equivalency and similarity were evaluated using fixed‐grid model‐cell permutation tests within the shared PCA‐env space (Broennimann et al. 2012). For the equivalency test, a one‐sided lower‐tail test was used to determine whether the observed Schoener's D was significantly lower than expected under the equivalency null hypothesis. Thus, *p* < 0.05 indicated significantly lower overlap than expected under equivalency and rejection of the equivalency null hypothesis. For the similarity test, the future density surface was randomly shifted within the corresponding environmental background according to environmental availability, and a one‐sided upper‐tail test was used to determine whether the observed Schoener's D was significantly greater than the randomized expectation. Thus, *p* < 0.05 indicated that current and future projected suitable environmental spaces were more similar than expected from environmental availability alone. Each test was performed with 1000 permutations. For each region, test type, and overlap metric, raw *p* values across the nine future scenarios were adjusted using the Holm procedure (Holm 1979), with adjusted *p* < 0.05 considered statistically significant. Projected suitable environmental‐space breadth was additionally characterized using the 95% KDE area, occupied PCA‐grid cells, and convex‐hull area. For each region–period combination, up to 50,000 predicted‐suitable raster cells were sampled with equal weights. A fixed axis‐specific bandwidth was estimated from the pooled balanced suitable‐cell sample using MASS::bandwidth.nrd and applied consistently across all combinations (Venables and Ripley 2002). KDE was evaluated on the common 100 × 100 PCA‐env grid, and grid cells were ranked from highest to lowest density (Broennimann et al. 2012; Di Cola et al. 2017). The 95% KDE area was defined as the area of the smallest set of grid cells containing 95% of the cumulative density and was expressed in squared PC units (Hyndman 1996). All analyses were conducted in R v4.5.2 using the “ecospat v4.1.4” and “mass” package.

### 2.5 Analysis of Global Multivariate Environmental Similarity Surface (MESS) and Most Dissimilar Variable (MoD)

Multivariate Environmental Similarity Surface (MESS) analysis was used to assess environmental novelty and potential extrapolation uncertainty in the G‐SDM projections under future scenarios (Elith et al. 2010). To ensure consistency with the G‐SDM, the terrestrial and marine MESS analyses were conducted separately using the final predictor sets retained in the corresponding G‐SDM components. Current environmental conditions within the G‐SDM calibration domain were used as the reference against which future conditions were evaluated. MESS values were calculated for each grid cell under each SSP scenario and time periods. Negative MESS values indicate that at least one predictor falls outside its reference range, whereas non‐negative values indicate that no predictor exceeds its corresponding univariate reference range (Elith et al. 2010). The most dissimilar variable (MoD) was identified for each grid cell as the predictor with the lowest environmental similarity score relative to the reference domain (Elith et al. 2010). Analyses were conducted in R using the “dismo” and “raster” packages.

## 3 Results

### 3.1 Model Evaluation

The TSS and AUC values obtained from random‐holdout validation indicated generally good model discrimination across the four geographic SDMs (Table 2). For the G‐SDM, the mean TSS and AUC were 0.85 and 0.94 for the terrestrial component and 0.83 and 0.87 for the nearshore component, respectively. The corresponding values for the N‐SDM were 0.81 and 0.88 for the terrestrial component and 0.85 and 0.91 for the nearshore component. For the NA‐SDM, the mean TSS and AUC were 0.86 and 0.92 for the terrestrial component and 0.82 and 0.88 for the nearshore component, respectively. For the EUR‐SDM, the corresponding values were 0.87 and 0.88 for the terrestrial component and 0.90 and 0.92 for the nearshore component. EMmean was selected for the terrestrial G‐SDM, N‐SDM, and NA‐SDM and for the nearshore G‐SDM and NA‐SDM, whereas EMmedian was selected for the terrestrial EUR‐SDM and the nearshore N‐SDM and EUR‐SDM.

**TABLE 2:** Validation‐set evaluation metrics from the repeated random‐holdout modeling procedure and selected ensemble methods for the four geographic species distribution models of *Eriocheir sinensis* .

Model	Validation TSS of land (mean)	Validation AUC of land (mean)	Validation TSS of nearshore (mean)	Validation AUC of nearshore (mean)	Best ensemble method (land)	Best ensemble method TSS	Best ensemble method (nearshore)	Best ensemble method TSS
G‐SDM	0.85 ± 0.07	0.94 ± 0.06	0.83 ± 0.13	0.87 ± 0.08	EMmean	0.87	EMmean	0.86
N‐SDM	0.81 ± 0.12	0.88 ± 0.09	0.85 ± 0.15	0.91 ± 0.09	EMmean	0.91	EMmedian	0.93
NA‐SDM	0.86 ± 0.07	0.92 ± 0.08	0.82 ± 0.17	0.88 ± 0.10	EMmean	0.84	EMmean	0.87
EUR‐SDM	0.87 ± 0.08	0.88 ± 0.09	0.90 ± 0.06	0.92 ± 0.09	EMmedian	0.89	EMmedian	0.82

*Note:* Values are presented as mean ± SD across algorithms and repeated cross‐validation runs.

In addition, spatial block cross‐validation was used to further assess the robustness of model discrimination under spatially separated validation conditions with respect to spatial autocorrelation (Figure S3). Mean ROC‐AUC values ranged from 0.77 to 0.94 and mean TSS values from 0.55 to 0.77 across the four geographic SDMs. Despite the decline in evaluation scores, the models retained moderate to good discriminatory ability under spatially separated validation.

### 3.2 Analysis of Variable Response

The relative importance of environmental predictors varied among the regional SDMs (Figure 3). In the N‐SDM, the five highest‐contributing predictors of terrestrial suitability were annual mean temperature (bio1), distance to shoreline (bio20), isothermality (bio3), mean temperature of the wettest quarter (bio8), and annual precipitation (bio12), in decreasing order of importance. In the EUR‐SDM, the five leading predictors were distance to shoreline (bio20), annual mean temperature (bio1), mean temperature of the wettest quarter (bio8), isothermality (bio3), and annual precipitation (bio12). In contrast, terrestrial suitability in the NA‐SDM was primarily associated with annual precipitation (bio12), annual mean temperature (bio1), and precipitation of the warmest quarter (bio18). For nearshore brackish‐water suitability, the main predictors were consistent across the three regional models, with water depth (M‐bio4), mean chlorophyll concentration (M‐bio15), mean sea surface temperature (M‐bio13), and mean salinity (M‐bio10) showing the highest contributions.

**FIGURE 3:**
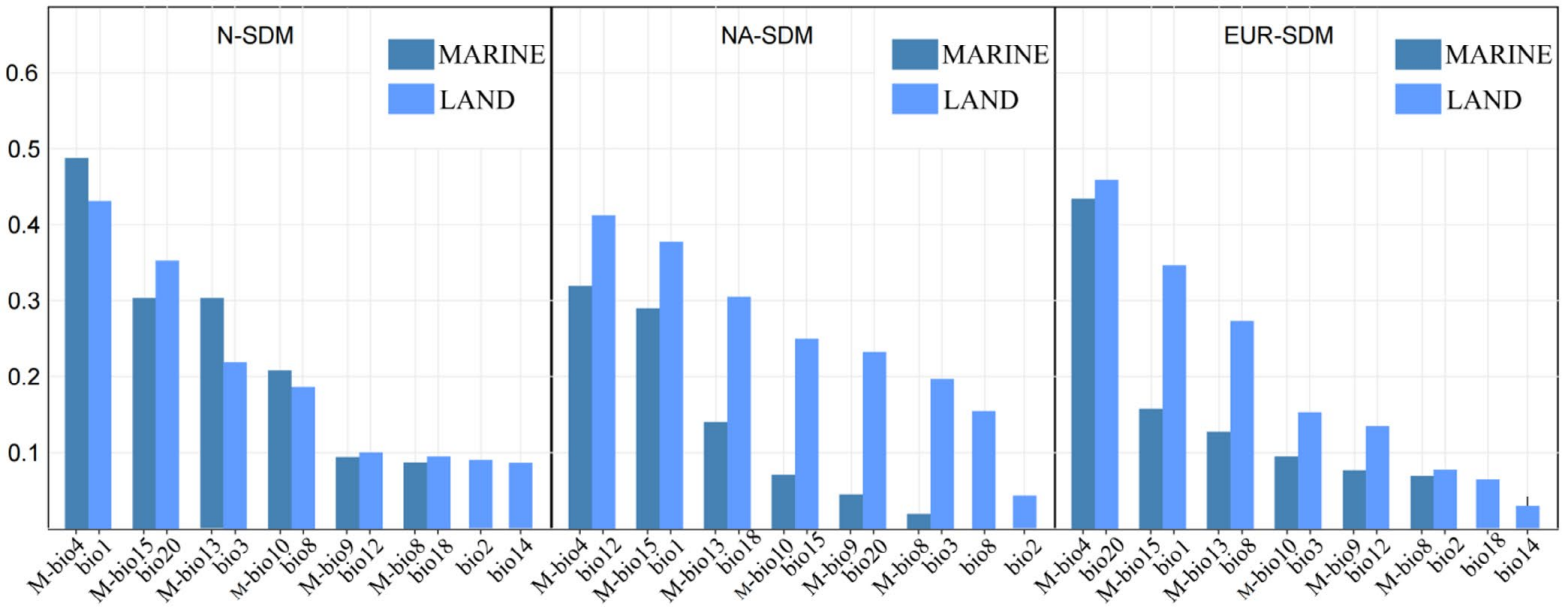
Importance of environmental predictors to the regional species distribution models of *Eriocheir sinensis* .

To compare the predicted environmental suitability of *E. sinensis* across regions, we analyzed four terrestrial bioclimatic variables and three marine environmental variables selected based on their contributions in the N‐SDM. Using region‐specific suitability thresholds (N‐SDM: 0.71; EUR‐SDM: 0.67; NA‐SDM: 0.64), the central 95% ranges of highly suitable terrestrial conditions differed among regions (Figures 4, 5). For bio01, bio03, bio08, and bio12, these ranges were 9.3°C–20.4°C, 20.9°C–29.4°C, 19.5°C–26.9°C, and 535–1576 mm in the native range; 4.3°C–15.7°C, 21.6°C–39.9°C, 3.5°C–19.5°C, and 460–1086 mm in Europe; and 3.8°C–15.4°C, 23.6°C–40.9°C, −4.3°C–24.0°C, and 744–1711 mm in North America. Thus, both invaded regions extended towards cooler temperature conditions, particularly for bio08. For nearshore brackish‐water suitability thresholds of 0.76, 0.74, and 0.79, respectively, the central 95% ranges of M‐bio13, M‐bio15, and M‐bio10 were 13.8°C–25.5°C, 0.66–6.33, and 11.8–31.3 in the native range; 7.3°C–18.7°C, 0.50–2.31, and 3.1–32.5 in Europe; and 7.0°C–24.5°C, 0.67–2.88, and 12.6–32.7 in North America (Figure 5). The invaded regions therefore included lower sea‐surface temperatures but narrower chlorophyll ranges than the native range.

**FIGURE 4:**
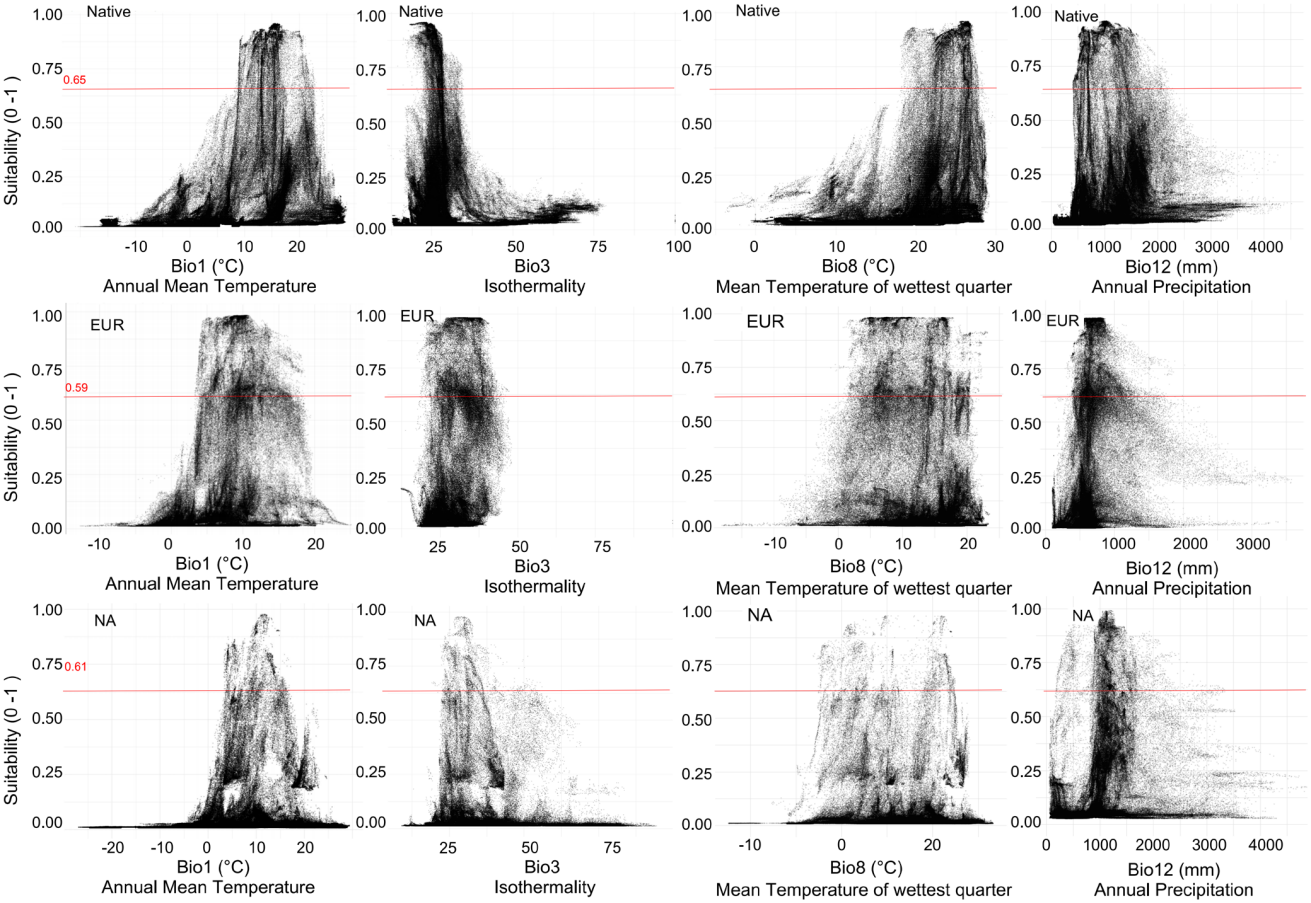
Modeled terrestrial suitability responses of *Eriocheir sinensis* across regions to the four highest‐ranking bioclimatic predictors identified by the native‐range model. Red horizontal lines indicate the region‐specific thresholds used to convert continuous suitability predictions into binary suitable‐unsuitable classifications.

**FIGURE 5:**
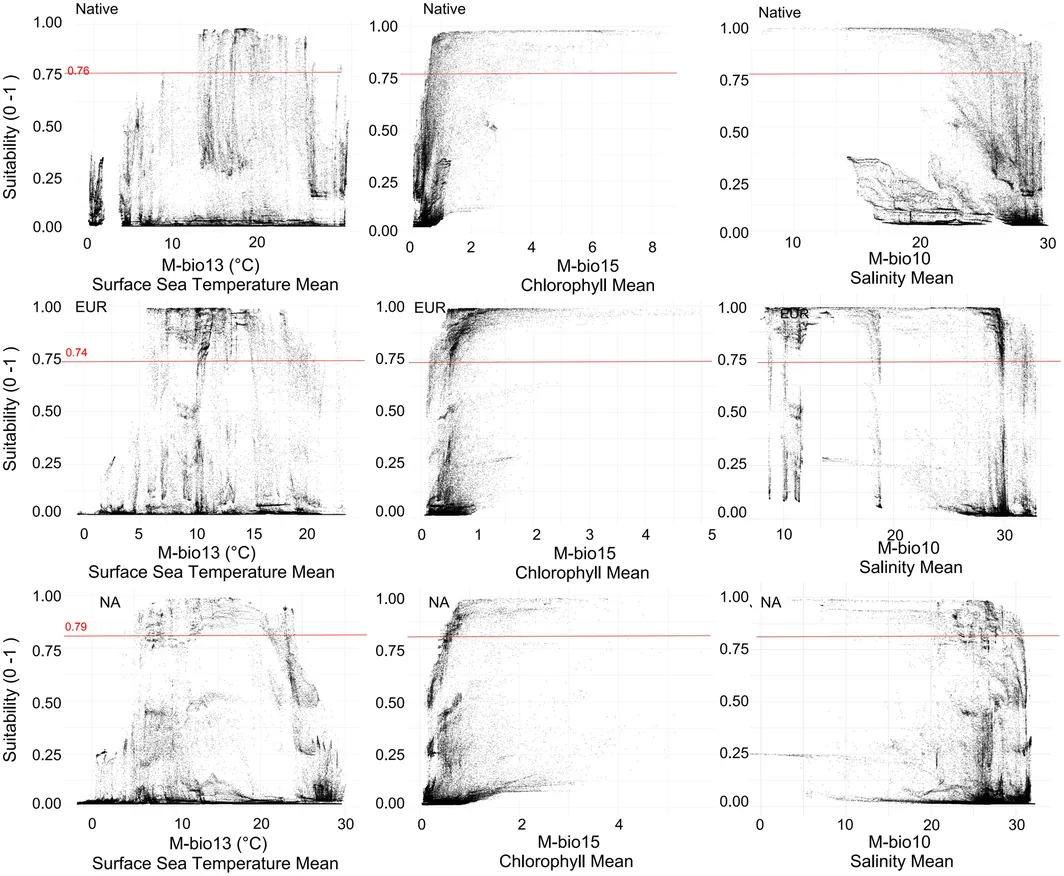
Modeled nearshore suitability responses of *Eriocheir sinensis* to selected important marine environmental predictors across regions. Predictors were selected based on their importance in the regional models. Red horizontal lines indicate the region‐specific thresholds used to convert continuous suitability predictions into binary suitable‐unsuitable classifications.

The G‐SDM was primarily used to characterize the global distribution and future changes in potential suitable habitats; therefore, comparisons of variable importance and suitability responses were restricted to the three independently calibrated regional models.

### 3.3 Current Potentially Suitable Habitats for *E. sinensis*

The N‐SDM predictions show that the potential suitable habitats within the native range of *E. sinensis* are mainly concentrated in the coastal regions between 27°N and 42°N, including the native distribution areas along the coast of Shandong, Liaoning, and Jiangsu provinces in China, and the coastal regions of the Korean Peninsula under the current climate scenario (Figure 7).

The NA‐SDM and EUR‐SDM projected that suitable areas for *E. sinensis* were concentrated along the Atlantic coast, around the Great Lakes, and in parts of the Pacific coast of North America, as well as along the North Sea, Baltic Sea, and Black Sea coasts of Europe (Figures 8, 9). The G‐SDM additionally projected potentially suitable areas around the Seto Inland Sea and Osaka Bay in Japan, along the eastern coast of Argentina, the southern coast of Brazil, and the coast of Uruguay, as well as along the southern coast of Australia (Figure 6).

**FIGURE 6:**
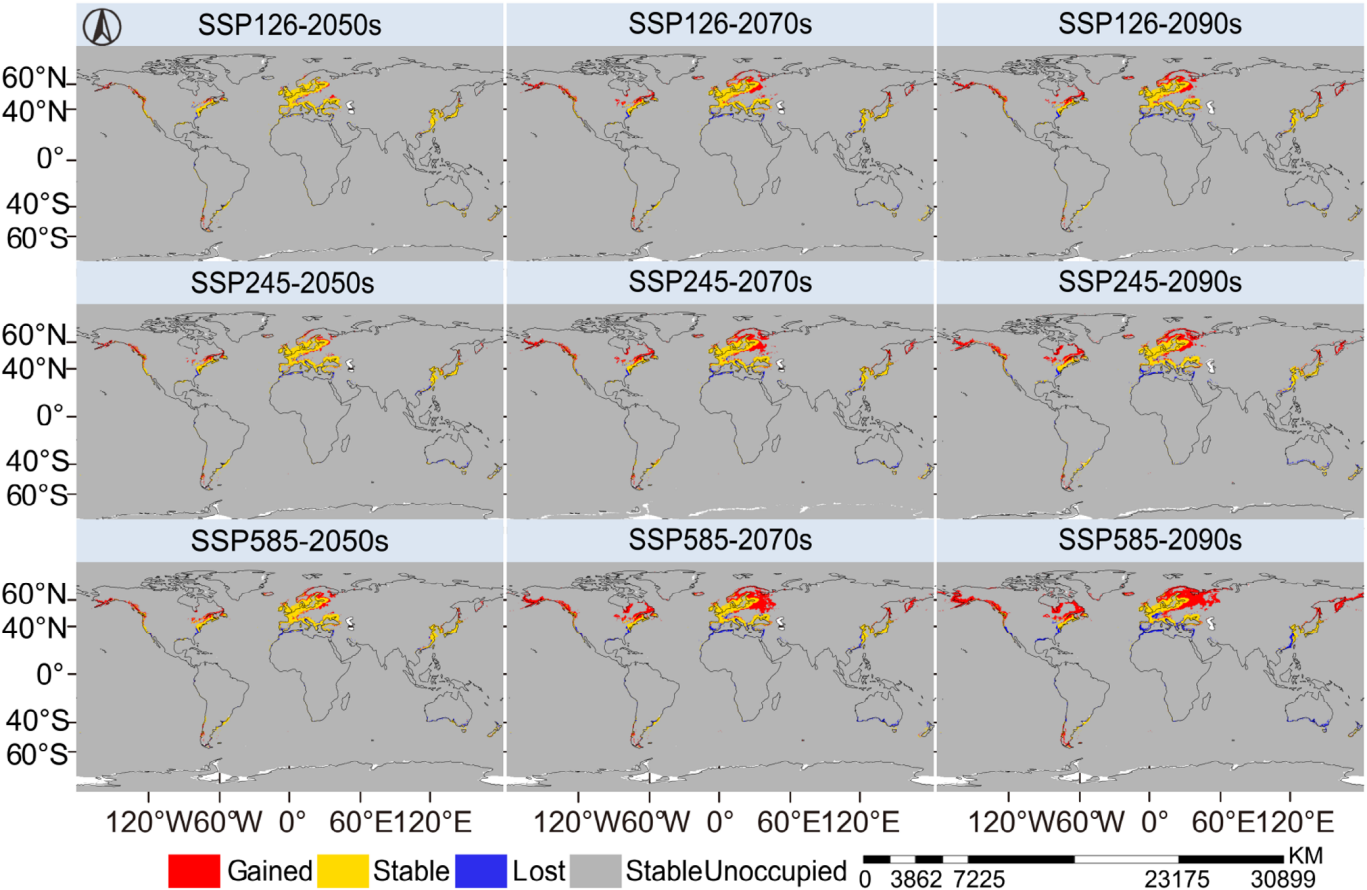
Future changes in potential suitable habitats under the G‐SDM were mapped by spatially combining the binary predictions of terrestrial and nearshore brackish‐water suitable areas and comparing future scenarios with current conditions. The binary classification thresholds were set at 0.71 for terrestrial habitats and 0.78 for nearshore brackish‐water habitats.

However, the terrestrial and nearshore sub‐model projections did not fully coincide across several parts of the potential global range. In some areas, suitable conditions were predicted by only one of the two independently fitted sub‐models. This limited spatial correspondence was particularly evident across much of Newfoundland in North America and along parts of the northern Mediterranean coast in Europe (Figures 8, 9).

### 3.4 Dynamic Changes in the Suitable Habitats for *Eriocheir sinensis* Under Future Climate Scenarios

N‐SDM projections for the native range showed relatively limited changes under SSP126, with reductions in predicted suitability occurring mainly across inland eastern China. Under the SSP245 and SSP585 scenarios, predicted suitability progressively increased in northeastern China, the Korean Peninsula, and other regions north of 40°N, but declined further across eastern and southern China. These changes were most pronounced under SSP585 in the 2090s, when areas of high predicted suitability in eastern China were nearly absent. Compared with terrestrial environments, predicted suitability in nearshore brackish‐water environments showed less pronounced changes across future climate scenarios (Figure 7). Nevertheless, marked declines were also projected along the eastern coast of China under SSP585.

**FIGURE 7:**
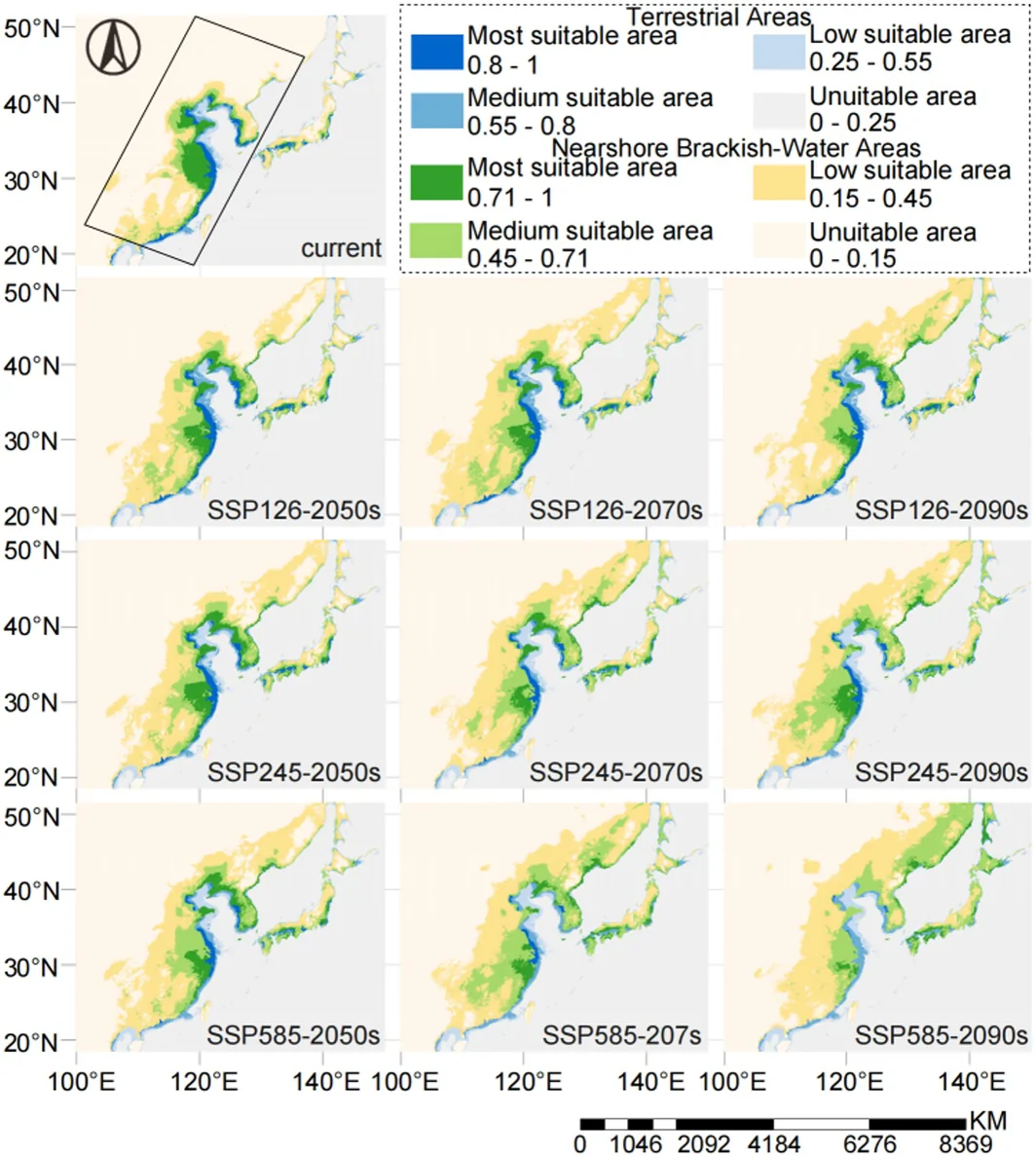
Model predictions for the native range of *E. sinensis* in the Asia‐Pacific coastal region (N‐SDM) under different climate change scenarios and time scales. Black boxes in the top panel indicate the main native distribution areas of *E. sinensis* in China and the Korean Peninsula.

EUR‐SDM projections showed relatively limited changes under SSP126, with predicted suitable areas remaining concentrated along the North Sea, Baltic Sea, Black Sea, and Atlantic coast of Western Europe (Figure 8). Under SSP245 and SSP585, terrestrial suitability progressively expanded northward and eastwards into Scandinavia and northern European Russia. This expansion was most pronounced under SSP585 in the 2070s and 2090s, when extensive suitable areas emerged north of 60°N. However, terrestrial and nearshore suitable areas did not shift synchronously: under later SSP2‐4.5 and SSP5‐8.5 scenarios, terrestrial suitability expanded into northern Scandinavia and northern European Russia without a corresponding expansion of nearshore brackish‐water suitability.

**FIGURE 8:**
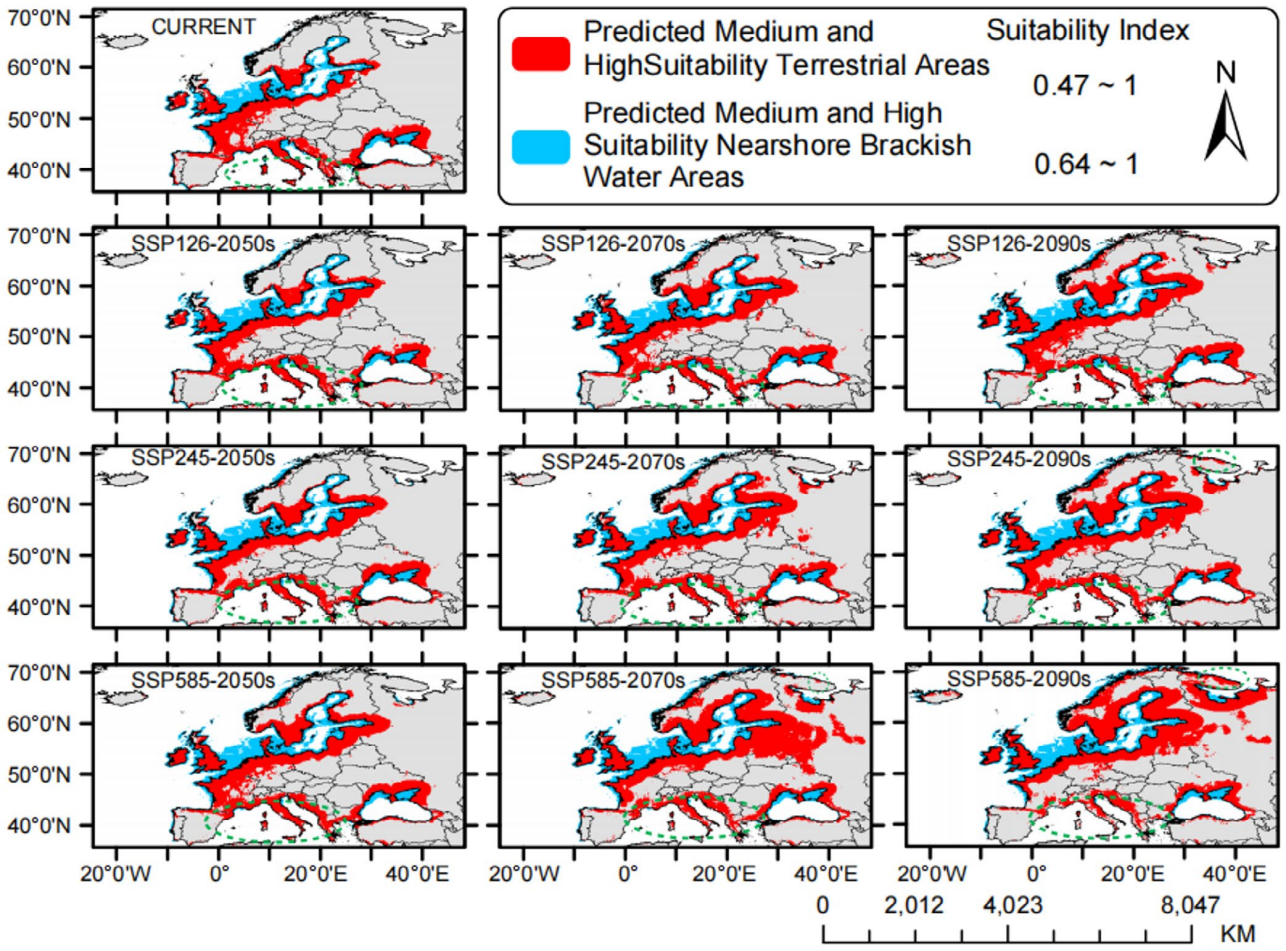
The prediction results of suitable habitats within the European range in the EUR‐SDM (For clarity, only areas of moderate and high predicted suitability are shown to highlight the spatial correspondence between terrestrial and nearshore suitable areas). Green circles indicate areas lacking corresponding suitable nearshore brackish‐water environments.

NA‐SDM projections likewise indicated an overall northward redistribution of terrestrial suitability (Figure 9). Changes were relatively limited under SSP126. Under SSP245 and SSP585, predicted suitability progressively increased across eastern Canada, with newly suitable areas occurring mainly around Newfoundland, Labrador, and the Gulf of St Lawrence, as well as in southern Alaska. The expansion was most pronounced under SSP585 in the 2070s and 2090s. Predicted suitable areas along the Atlantic and Pacific coasts were largely retained, whereas spatial changes in nearshore brackish‐water suitability were generally less pronounced than those in terrestrial freshwater suitability. In particular, future terrestrial suitable areas gradually expanded into eastern and northeastern Canada, including Newfoundland and Labrador and areas farther north, but some newly suitable terrestrial areas were not accompanied by corresponding suitable nearshore brackish‐water areas.

**FIGURE 9:**
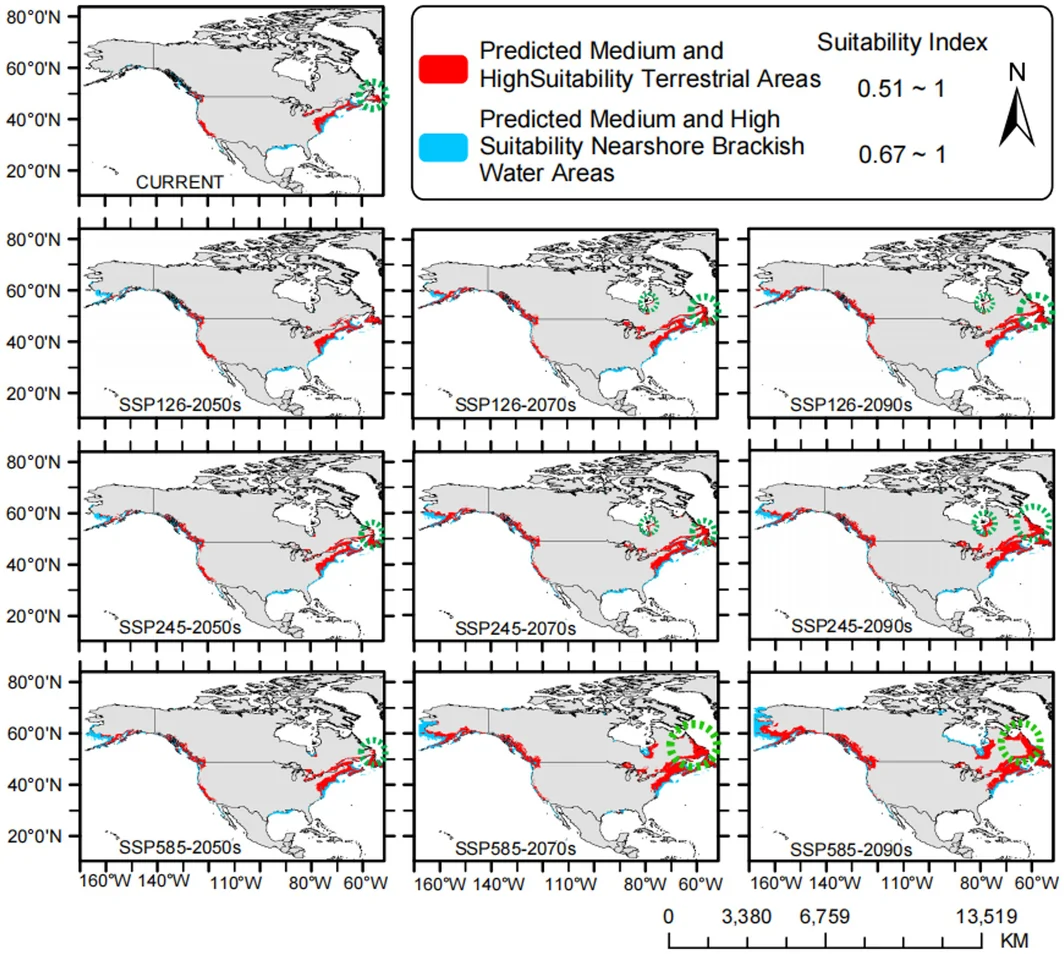
The prediction results of suitable habitats in North America within the NA‐SDM (For clarity, only areas of moderate and high predicted suitability are shown to highlight the spatial correspondence between terrestrial and nearshore suitable areas). Green circles indicate areas lacking corresponding suitable nearshore brackish‐water environments.

G‐SDM projections indicated an overall poleward redistribution of predicted suitable areas for *E. sinensis* under future climate scenarios (Figure 6). Areas remaining suitable were retained mainly in western and central Europe, eastern North America, and East Asia. Changes under SSP126 were relatively limited. By the 2090s, however, newly suitable areas had emerged in northern Europe, northern North America, and the Russian Far East. Under SSP245 and SSP585, gains at high northern latitudes became progressively more extensive over time. In contrast, losses occurred mainly along the lower‐latitude margins of the current predicted suitable range, with pronounced reductions in southern Australia and along the southern Mediterranean coast.

### 3.5 Changes in Projected Suitable Environmental Space Under Future Climate Scenarios

A common PCA calibrated from the current environmental backgrounds of the three regions explained 70.0% of the total environmental variation, with PC1 and PC2 accounting for 42.6% and 27.4%, respectively (Figure 10). Schoener's D was 0.435 between the native range and Europe and 0.417 between the native range and North America. Both comparisons showed significantly greater overlap than expected from environmental availability alone in the similarity test (*p* < 0.05), whereas the equivalency test did not reject the null hypothesis of equivalency.

**FIGURE 10:**
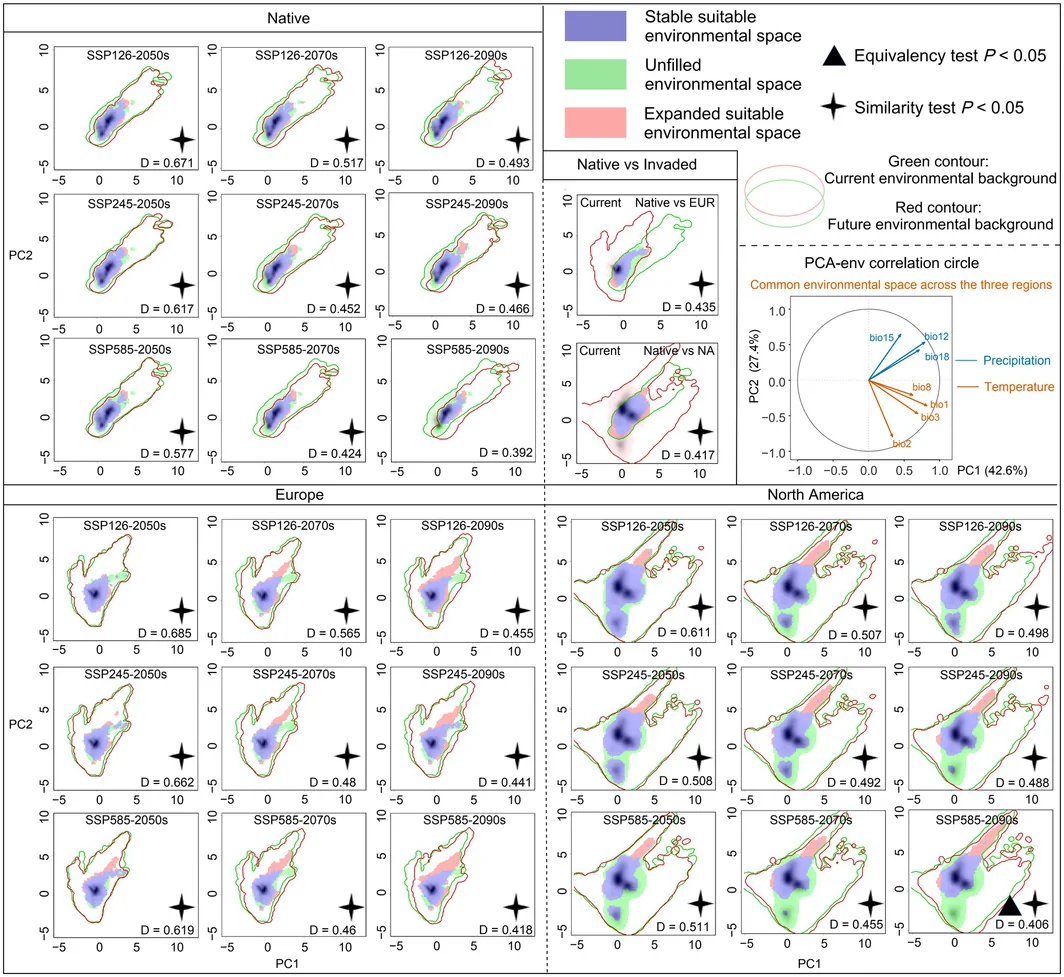
PCA‐env comparisons of predicted suitable environmental space for *Eriocheir sinensis* within and among geographic regions. Current and future predictions for the native range, Europe, and North America were projected into a shared PCA‐env space calibrated from the current environmental backgrounds of the three regions. Expansion indicates newly suitable environmental space under future conditions, stability indicates suitable environmental space retained between current and future conditions, and unfilling indicates current suitable environmental space that is no longer represented under future conditions.

Within each region, overlap between current and future predicted suitable environmental spaces generally decreased towards later periods and under higher‐emission scenarios (Figure 10). Across the three SSPs, Schoener's D declined from 0.671–0.577 in the 2050s to 0.493–0.392 in the 2090s for the native range, from 0.685–0.619 to 0.455–0.418 for Europe, and from 0.611–0.508 to 0.498–0.406 for North America. The lowest overlap in all three regions occurred under SSP585 in the 2090s. Similarity tests were significant for most current–future comparisons, indicating greater similarity than expected from environmental availability alone; the only exception was the native‐range comparison under SSP585 in the 2090s. In addition, the equivalency test rejected the null hypothesis of equivalency for North America under SSP585 in the 2090s.

The environmental‐space expansion index remained low across all regions and climate scenarios, ranging from 0.002 to 0.027 (0.2%–2.7%) (Figure 11). In contrast, the unfilling index generally increased towards later periods and under higher‐emission scenarios. By the 2090s, unfilling across SSP126–SSP585 ranged from 0.032 to 0.170 in the native range and from 0.063 to 0.237 in North America, compared with only 0.018–0.042 in Europe.

**FIGURE 11:**
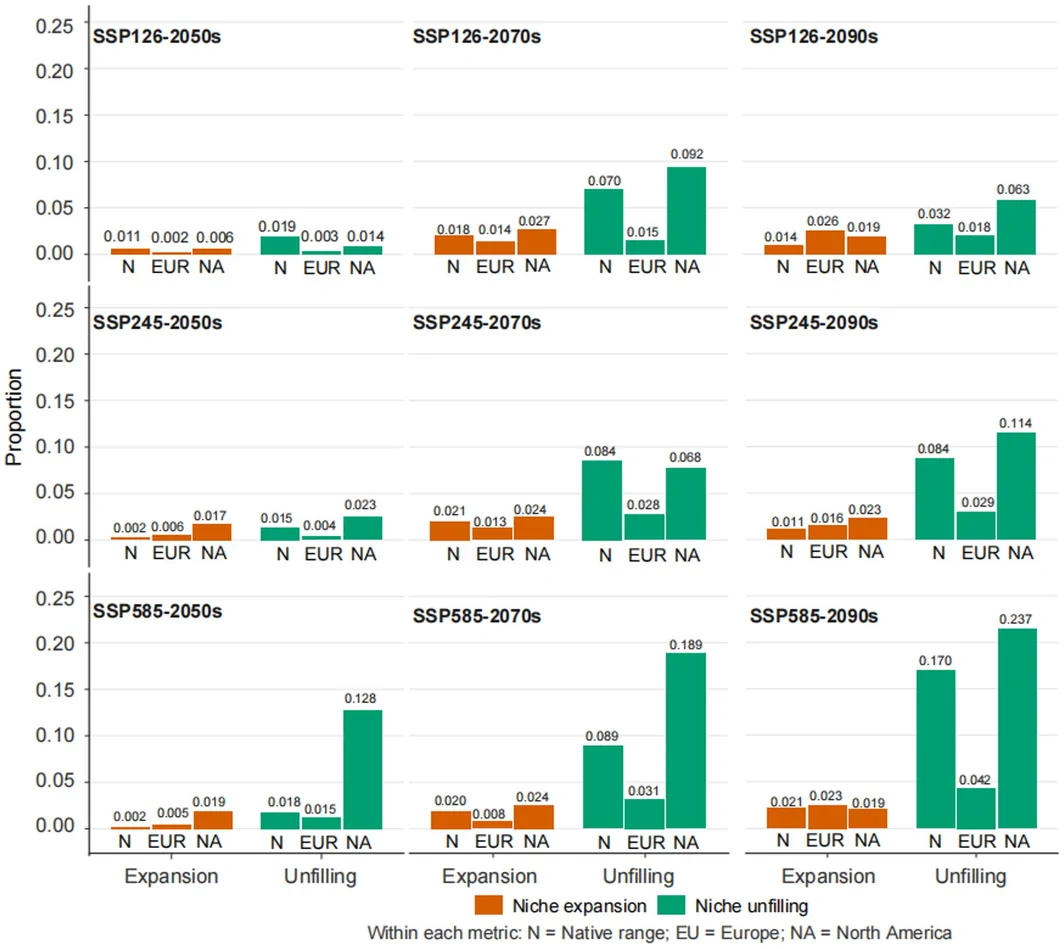
Projected terrestrial environmental‐space dynamics of *Eriocheir sinensis* across its native range, Europe, and North America under nine future climate scenarios. Environmental‐space expansion represents the proportion of the future predicted‐suitable environmental density occurring outside the current suitable environmental space, whereas Environmental‐space unfilling represents the proportion of the current suitable environmental density not covered under future conditions.

The three metrics revealed contrasting trajectories in predicted suitable environmental space among regions (Figure 12). The native range showed an overall contraction, with the 95% KDE area, occupied PCA‐grid cells, and convex‐hull area decreasing from approximately 6.6 PC units^2^, 390 cells, and 16.5 PC units^2^ under current conditions to 4.1 PC units^2^, 235 cells, and 10.7 PC units^2^ under SSP585‐2090s, respectively. Europe showed comparatively stable KDE breadth but broader occupied‐cell and convex‐hull extents under many future scenarios, with occupied‐cell counts and convex‐hull areas exceeding current values under most future scenarios and peaking at approximately 575 cells and 24.3 PC units^2^ under SSP126‐2090s. North America had the broadest environmental space under current conditions, but all three metrics generally declined over time. By SSP585‐2090s, its 95% KDE and convex‐hull areas had decreased from approximately 9.2 and 23.0 to 6.3 and 16.0 PC units^2^. Overall, pronounced contractions were projected for the native range and North America under the late high‐emission scenario, whereas Europe remained comparatively stable and showed some expansion.

**FIGURE 12:**
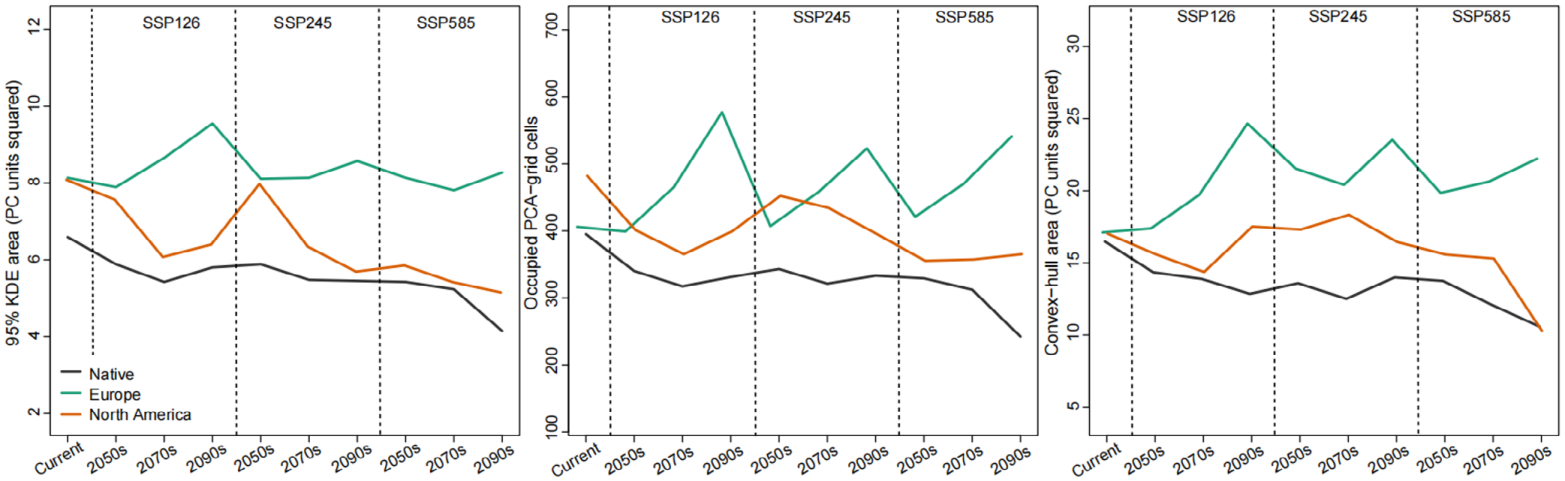
Predicted suitable environmental‐space breadth of *Eriocheir sinensis* across its native range, Europe, and North America under current and future climate scenarios. All metrics were calculated in a common PCA‐env space calibrated using balanced samples from the current environmental backgrounds of the three regions. Vertical dotted lines separate the SSP126, SSP245, and SSP585 scenarios. Lines connect scenario‐specific estimates for visual comparison and do not represent fitted temporal trends. KDE and convex‐hull areas are expressed in squared PC units.

### 3.6 Global Analysis of Multivariate Environmental Similarity Surface (MESS) and the Most Dissimilar Variable (MoD)

Spatial comparison of the global MESS and G‐SDM projections revealed marked geographic variation in environmental novelty within and around the predicted suitable areas of *E. sinensis* (Figures 6, 13). Negative MESS values occurred mainly in Western Europe and along the North Sea coast; along the Pacific coast of North America, the Atlantic coast of northeastern North America, and the northern Gulf of Mexico; and across the Korean Peninsula, Japan, and southern coastal China. Some areas predicted as suitable by the G‐SDM were located within these negative‐MESS regions, indicating that the corresponding suitability projections involved at least one variable outside its present‐day reference range and were therefore subject to extrapolation uncertainty.

**FIGURE 13:**
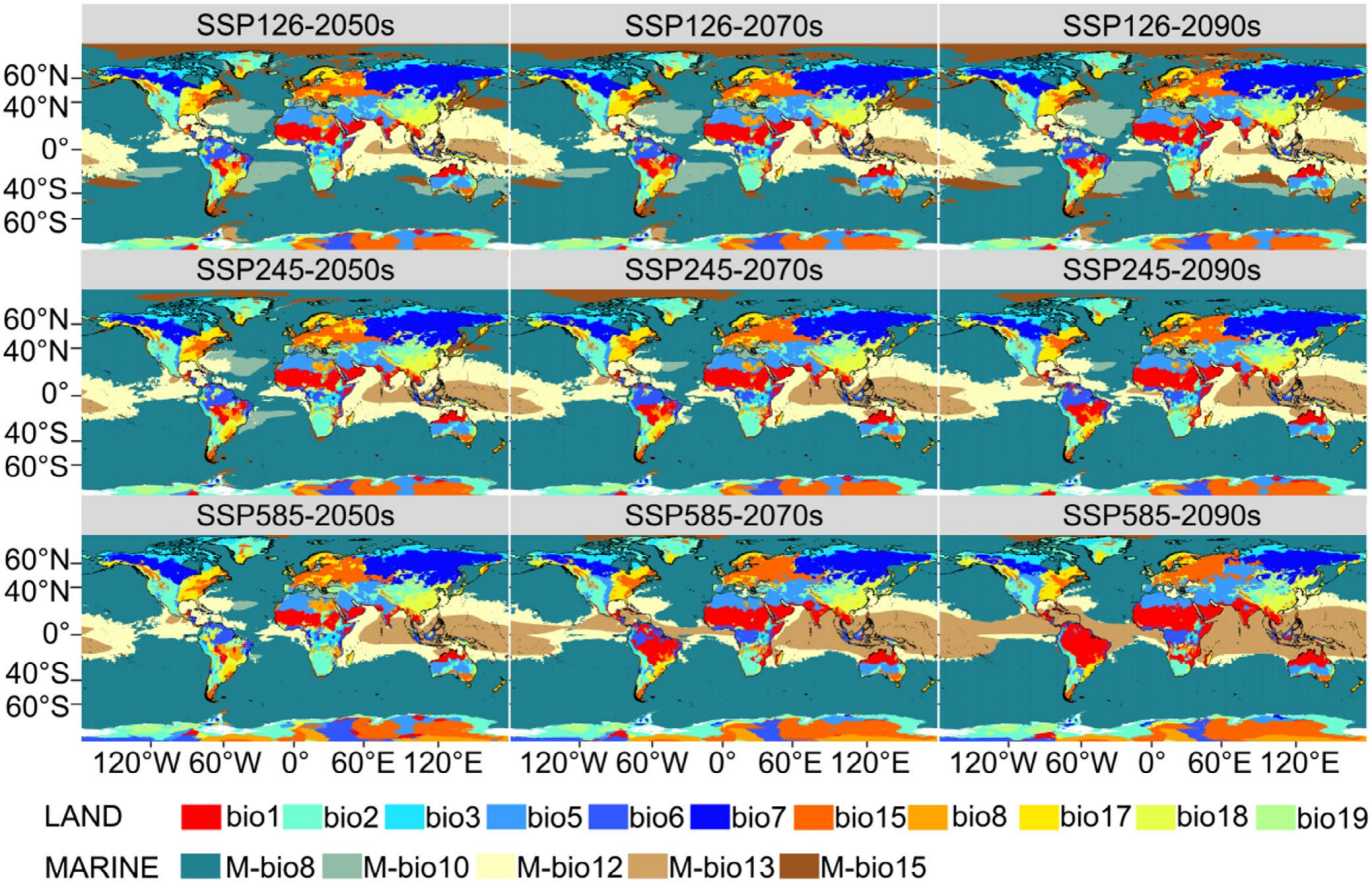
Global multivariate environmental similarity surface (MESS) under future climate scenarios. MESS was calculated using the environmental predictors retained in the corresponding terrestrial and nearshore components of the G‐SDM, relative to the current reference domain.

The spatial distribution of MoD varied among regions and emission scenarios (Figure 14). In terrestrial environments, precipitation seasonality (bio15) was most frequently identified as the MoD in parts of Europe. Precipitation of the coldest quarter (bio19) occurred predominantly as the MoD along the Pacific coast of North America, annual temperature range (bio7) along the Atlantic coast of northeastern North America, and precipitation of the warmest quarter (bio18) in East Asia. In nearshore marine environments, the composition of MoD variables changed across emission scenarios. In Europe, the principal MoD variables shifted from salinity (M‐bio10) and chlorophyll concentration (M‐bio15) under SSP126 to salinity and pH (M‐bio8) under SSP585. Along the Pacific coast of Asia, they shifted from salinity and chlorophyll concentration to pH and maximum sea‐surface temperature (M‐bio12), whereas in North America, they shifted from chlorophyll concentration and maximum sea‐surface temperature to pH and maximum sea‐surface temperature.

**FIGURE 14:**
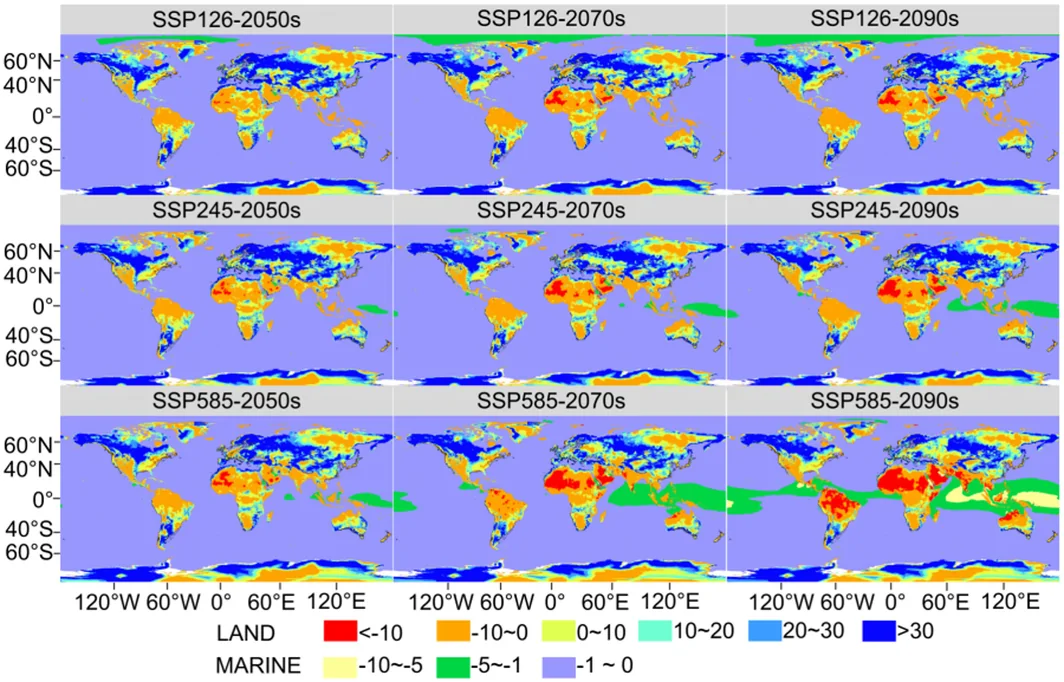
Global distribution of the most dissimilar variable (MoD) under future climate scenarios.

## 4 Discussion

### 4.1 Model Performance and Environmental Associations

The four geographic SDMs showed generally good model‐fitting performance. However, pseudo‐absences represent background locations rather than confirmed absences, and their selection can affect model evaluation and spatial predictions (Barbet‐Massin et al. 2012; VanDerWal et al. 2009). Particularly in the G‐SDM, pseudo‐absences sampled across a broad geographic extent may have included environmentally distinct or not necessarily accessible areas, increasing the contrast with occurrence records and potentially producing optimistic performance estimates. Consistent with this possibility, spatial block cross‐validation yielded lower scores than random holdout validation, with mean ROC‐AUC values of 0.77–0.94 and TSS values of 0.55–0.77 across the four geographic SDMs (s Figure S3). Nevertheless, the models retained moderate to good discriminatory ability under spatially separated validation, suggesting that model discrimination was not solely attributable to spatial dependence between calibration and validation data. Coastal records may also be subject to some terrestrial–nearshore classification uncertainty due to raster resolution, coastline representation, and positional errors in occurrence coordinates. In addition, the omission of dispersal constraints and biotic interactions may result in predicted suitability in areas that are not actually occupied by the species (Araújo and New 2007; Guisan and Thuiller 2005). These evaluation results therefore support the broad‐scale characterization of environmental suitability but should not be interpreted as independent evidence of local occurrence or temporal transferability.

The regional models identified temperature, precipitation, and distance to the coastline as important predictors of terrestrial suitability for *E. sinensis* , although their relative contributions differed markedly among regions. Temperature‐related variables (bio1, bio3, and bio8) and distance to the coastline (bio20) were particularly important in the native range and Europe. The prominence of temperature is biologically plausible given its influence on the development and growth of *E. sinensis* , whereas the importance of distance to the coastline is consistent with its catadromous life cycle, in which prolonged freshwater residence is followed by downstream reproductive migration to estuarine and saline environments (Keirsebelik et al. 2025; Rudnick and Resh 2005). In contrast, precipitation‐related variables (bio12) contributed more strongly in North America, suggesting that they may capture regional hydroclimatic variation in freshwater availability and river flow rather than direct physiological constraints.

Compared with the native range, conditions associated with high predicted suitability in Europe and North America extended to lower annual mean temperatures and lower wettest‐quarter temperatures (Figure 4), indicating clear regional differences in modeled climatic associations. These differences may partly reflect variation in regional climatic backgrounds. Europe and North America include climatic conditions that are less well represented in the native‐range calibration environment, allowing regional occurrence records to capture colder environmental combinations. Environmental filtering may further restrict established populations to locally available climatic conditions compatible with their life‐cycle requirements. The broader suitable ranges estimated for several variables in Europe may also reflect the wider environmental gradients represented by its occurrence records. By contrast, the more limited geographic and environmental coverage of occurrence records in North America may contribute to narrower estimates of suitable environmental ranges for some variables, consistent with the potential effects of niche truncation in regionally calibrated SDMs (Chevalier et al. 2022). These differences are therefore better interpreted as modeled environmental associations shaped by environmental availability, range occupancy, and environmental filtering, rather than as evidence of climatic adaptation or expanded physiological tolerance. Predictor contributions were more consistent among the nearshore sub‐models, with water depth, chlorophyll concentration, sea‐surface temperature, and salinity consistently important (Figures 3, 5). Together, these variables characterize nearshore habitat structure, productivity, and physicochemical conditions. Temperature and salinity are particularly important during the early life stages of *E. sinensis* : both affect larval development and survival, while salinity also influences embryonic performance (Anger 1991; Wang et al. 2019). This is consistent with the species' catadromous life cycle, in which reproduction and larval development occur in estuarine or saline environments (Anger 1991; Zhang et al. 2019a). The contrast between terrestrial and nearshore predictors therefore indicates that the two sub‐models capture complementary environmental dimensions. Considering both projections can therefore help identify where suitable freshwater and nearshore brackish‐water environments coincide, without implying that hydrological connectivity or dispersal pathways were modeled.

### 4.2 Dynamics of *E. sinensis* Suitable Habitats Under Climate Change

Future projections indicated an overall northward redistribution of suitable environmental conditions for *E. sinensis* (Figure 6), consistent with the poleward shift of climatic conditions under ongoing warming (Burrows et al. 2014; Sunday et al. 2012). Temperature strongly affects juvenile growth, feeding, and molting, larval development, and life‐history timing in *E. sinensis* (Anger 1991; Rudnick and Resh 2005). Continued warming may therefore shift climatic conditions associated with relatively high suitability towards higher latitudes, while conditions at the lower‐latitude margins of currently suitable areas may increasingly diverge from the current suitable climatic space. This is consistent with projected suitability gains at higher latitudes across the native range, Europe, and North America, together with declines in eastern and southern China, the southern Mediterranean, and southern Australia. Importantly, these changes did not represent a simple overall expansion of potential suitability. Instead, concurrent gains at higher latitudes and losses at lower latitudes indicated a spatial reconfiguration of suitable environmental conditions. The magnitude and direction of such shifts can vary with regional climate trajectories and geographic constraints (Burrows et al. 2014). Regional differences in the magnitude and direction of these changes may also reflect the current climatic context of each region (Chevalier et al. 2022; Guisan et al. 2014). Areas corresponding to the cooler portion of the modeled suitable climatic space may become more suitable as temperatures rise, whereas those near its warmer margin may become increasingly dissimilar from conditions currently associated with high suitability (Sunday et al. 2012). The contrasting trajectories of Europe, North America, and the native range therefore more likely reflect differences in climate exposure and environmental availability than climatic adaptation among regional populations. These patterns were most pronounced in the later, higher‐emission projections, particularly under SSP585.

Owing to its catadromous life cycle, *E. sinensis* uses freshwater and estuarine–nearshore environments during different life‐history stages (Keirsebelik et al. 2025; Rudnick and Resh 2005; Shen et al. 2023). Consequently, suitability in either realm alone may not fully represent the environmental conditions relevant across the species' life cycle (Hermoso et al. 2021; Shen et al. 2023). From this perspective, the terrestrial and nearshore sub‐models represent complementary environmental dimensions, and their spatial correspondence can be used to characterize the geographic co‐occurrence of conditions potentially relevant to different life‐history stages. Some regions were predicted as suitable by only one sub‐model but lacked corresponding suitability in the other. In this study, projected changes in terrestrial and nearshore suitability were spatially discordant, with terrestrial suitability often emerging earlier in newly suitable regions than corresponding nearshore brackish‐water suitability (Figures 8, 9). Considering both projections can therefore distinguish areas where suitable terrestrial and nearshore conditions co‐occur from areas suitable in only one realm. This distinction may help identify areas requiring further investigation of river‐network connectivity, reproductive habitats, and establishment potential (Hermoso et al. 2021; Shen et al. 2023). However, the overlay describes only the geographic correspondence between two independently fitted models. It does not show that spatial discrepancies impose reproductive constraints or predict hydrological connectivity, dispersal pathways, or establishment success, because the relevant processes and confirmed spawning or nursery sites were not incorporated into the models (Jarnevich et al. 2015). Moreover, because the terrestrial and marine datasets used different current baseline periods, their overlap should be interpreted as complementary information on life‐history‐related suitability rather than as strictly contemporaneous terrestrial–marine environmental correspondence.

MESS analysis showed that some future suitable areas projected by the G‐SDM contained environmental conditions outside the current calibration range (Figure 13), indicating environmental extrapolation and greater uncertainty in these areas (Elith et al. 2010; Owens et al. 2013). Negative MESS values indicated that at least one predictor fell outside the range represented in the current calibration domain. They occurred mainly in Western Europe and along the North Sea coast, parts of coastal North America, and coastal East Asia, with some overlapping areas projected to gain or retain suitability. The three separately calibrated regional models provide complementary regional‐scale comparisons. Concordance between a regional model and the G‐SDM suggests that a broad spatial pattern is not unique to the global calibration, but it does not constitute independent validation or eliminate extrapolation uncertainty (Wenger and Olden 2012; Yates et al. 2018). MoD results further showed marked regional and scenario‐specific differences in the environmental dimensions with the greatest dissimilarity from current conditions (Figure 14). In terrestrial environments, the greatest dissimilarities mainly involved temperature‐ and precipitation‐related variables. In nearshore environments, the most dissimilar variables shifted from salinity and chlorophyll concentration towards pH and maximum sea‐surface temperature across future scenarios. Thus, both the geographic pattern and environmental dimensions of extrapolation varied across future scenarios. Importantly, MoD identifies the most dissimilar variable relative to the current calibration domain, not a causal driver of future suitability changes (Elith et al. 2010). Areas with negative MESS values, particularly those overlapping projected suitability gains, therefore warrant further evaluation using independent occurrence records, regional environmental data, or process‐based models.

### 4.3 Regional Dynamics of Projected Suitable Environmental Space

Because PCA‐env included only terrestrial predictors, the following patterns refer specifically to terrestrial suitability. Europe showed relatively high retention of projected suitable environmental space under future climate scenarios (Figure 11). Although Schoener's D generally declined over time, indicating reduced current–future overlap, Europe consistently showed the lowest unfilling among the three regions, reaching only 0.018–0.042 in the 2090s. Similarity tests further showed that current–future overlap remained greater than expected from regional environmental availability alone. Meanwhile, the 95% KDE area remained relatively stable (Figure 12), whereas occupied PCA‐grid cells and convex‐hull area increased under several scenarios despite consistently low Expansion. Taken together, these patterns suggest substantial retention of the current suitable environmental space, with changes mainly in its peripheral extent rather than simple expansion or contraction. This pattern may partly reflect Europe's broad environmental gradients and the relatively wide range of conditions represented as suitable by the EUR‐SDM (Atwater et al. 2018; Chevalier et al. 2022; Guisan et al. 2014). The longer non‐native history of *E. sinensis* in Europe provides relevant biogeographical context (Rudnick and Resh 2005), but the present analysis cannot attribute this pattern to residence time, environmental availability, or model calibration.

North America showed a contrasting trajectory of stronger contraction and reorganization. Its current projected suitable environmental space was the broadest among the three regions across all breadth metrics, but this pattern did not persist under future scenarios (Figure 11). The 95% KDE area and the number of occupied PCA‐grid cells generally declined (Figure 12), whereas the convex‐hull area fluctuated under some low‐ and intermediate‐emission scenarios but contracted markedly in the later period under SSP585. Expansion remained low, whereas Unfilling generally increased with time and emission level, reaching 0.063–0.237 in the 2090s. Under SSP585 in the 2090s, the equivalency test rejected the null hypothesis of equivalency after Holm correction. Nevertheless, the similarity test indicated that current–future overlap remained greater than expected from regional environmental availability alone. This pattern therefore indicates contraction and redistribution of current suitable environmental space while retaining substantial current–future overlap. It primarily reflects changes in future regional environmental conditions relative to the current suitable environmental space identified by the NA‐SDM. North America's shorter invasion history and its relatively geographically fragmented documented occurrence provide relevant biogeographical context (Rudnick and Resh 2005; Ruiz et al. 2006). However, because the PCA‐env analysis was based directly on binary SDM projections, these factors cannot be regarded as direct causes of the projected environmental‐space dynamics.

Within the predefined native‐range analysis domain, the projected suitable environmental space also contracted, particularly under late high‐emission conditions (Figures 10, 11). All three breadth metrics generally declined, expansion remained low, and unfilling increased substantially, indicating reduced retention of currently suitable environmental space. Schoener's D also declined overall and reached its lowest value under SSP585 in the 2090s. Under this scenario, the similarity test was not significant, indicating that current–future overlap was not greater than expected from regional environmental availability alone (Broennimann et al. 2012). This pattern was consistent with the geographical projections showing declining suitability in eastern and southern China and increasing suitability at higher latitudes. Together with the geographic projections, these results indicate a northward redistribution of suitable conditions within the native‐range domain (Burrows et al. 2014; Sunday et al. 2012). This apparent contraction must nevertheless be interpreted in relation to the predefined geographic extent of the N‐SDM. Because N‐SDM projections were restricted to the predefined native‐range domain, suitable conditions beyond its northern boundary could not be represented in the environmental‐space metrics. The results therefore indicate reduced retention and reorganization within the defined native‐range domain, but not contraction across a broader geographic extent (Anderson and Raza 2010; Chevalier et al. 2021).

Schoener's D between the native range and Europe and North America was 0.435 and 0.417 (Figure 10), respectively, indicating partial environmental‐space overlap. Similarity tests showed that both observed overlaps were significantly greater than expected under regional‐background randomization conditional on environmental availability (Broennimann et al. 2012; Warren et al. 2008). The non‐overlapping components may partly reflect regional environmental differences, as Europe and North America include climatic conditions less well represented in the native‐range domain (Guisan et al. 2014). Although the equivalency tests did not reject the null hypothesis, this should not be interpreted as evidence that the regional environmental spaces were identical (Broennimann et al. 2012; Warren et al. 2008). Overall, the native and invaded regions shared some modeled environmental associations, while also exhibiting distinct components of projected suitable environmental space that may partly reflect regional differences in environmental availability. The three regions exhibited distinct trajectories of projected suitable environmental‐space change. Europe showed relatively high retention of its current projected suitable environmental space, whereas North America exhibited more pronounced contraction and redistribution under late high‐emission conditions. Within its predefined projection domain, the native range showed reduced environmental‐space retention, consistent with the northward redistribution in the geographic projections (Anderson and Raza 2010; Guisan et al. 2014; Yates et al. 2018). These differences may reflect regional environmental availability, future climate exposure, current suitability patterns, and the extent of the projection domains. These patterns reflect regional reorganization of projected suitable environmental space rather than direct evidence of niche evolution, adaptive divergence, or altered physiological tolerance (Kearney and Porter 2009; Soberón and Nakamura 2009).

### 4.4 Management Implications and Future Directions

The terrestrial and nearshore projections together provide a basis for monitoring that considers the life‐history requirements of *E. sinensis* (Hermoso et al. 2021; Rudnick and Resh 2005). Areas where predicted suitable freshwater and nearshore brackish‐water environments occur in close proximity may warrant particular attention because they contain conditions potentially relevant to different stages of the catadromous life cycle (Keirsebelik et al. 2025; Shen et al. 2023). River‐network post‐processing further identified candidate areas in Europe and North America where river‐associated terrestrial suitability spatially coincided with nearshore suitability (Figure 15). Estuaries and river–sea transition zones within these areas could therefore be prioritized for field validation, early detection, and continued surveillance (Cook et al. 2025). However, these locations represent candidate areas identified through spatial screening rather than confirmed reproductive or establishment hotspots. The ecological relevance of this spatial correspondence should be further evaluated using hydrological connectivity, dispersal conditions, biotic interactions, and verified reproductive sites to determine whether these areas can support multiple life‐history stages of *E. sinensis* (Guisan and Thuiller 2005; Robinson et al. 2011).

**FIGURE 15:**
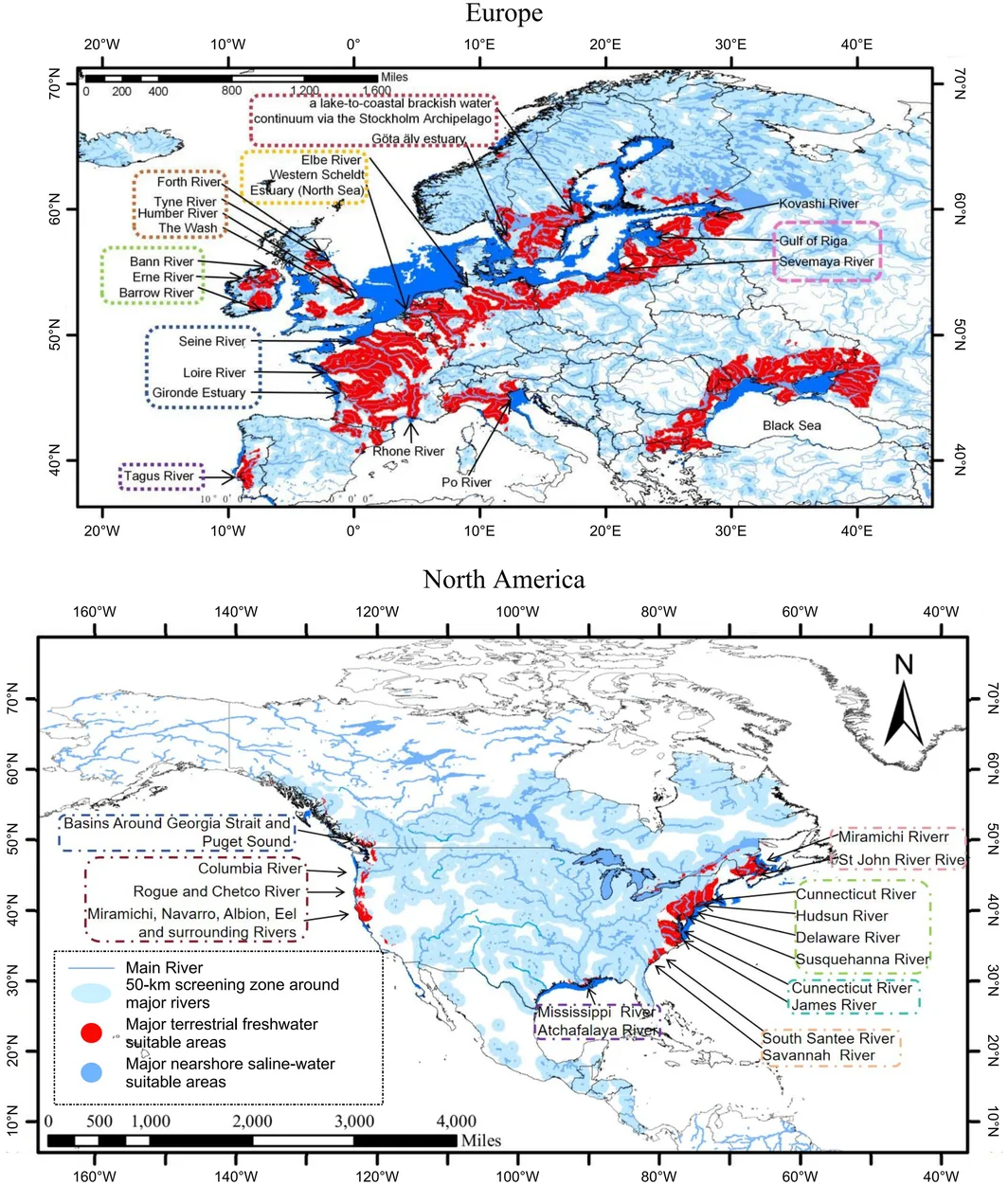
Schematic of river network‐based habitat connectivity mask and selection results of brackish and fresh water suitable habitat for *E. sinensis* . River‐network‐based spatial screening was conducted to identify candidate areas in Europe and North America where predicted suitable terrestrial freshwater habitats, selected river networks, and predicted suitable nearshore brackish‐water habitats were geographically aligned. The river buffers represented predefined post‐processing analysis zones and should not be interpreted as direct estimates of the species' actual dispersal distance or hydrological connectivity.

Regional management priorities should account for the contrasting trajectories of projected suitable environmental space. In Europe, the relatively high retention of currently suitable environmental conditions supports continued surveillance of established river–estuary systems and areas where suitability is projected to persist (Dittel and Epifanio 2009; Herborg et al. 2003). Monitoring of interconnected waterways and transport pathways may also help reduce secondary spread. In North America, the projected contraction and redistribution of suitable environmental space under late high‐emission conditions indicate that surveillance should not be restricted to currently documented locations. Greater attention may therefore be warranted in higher‐latitude coastal and river‐associated areas where terrestrial and nearshore suitability persists or emerges.

Within the predefined native‐range domain, declining environmental‐space retention and northward redistribution suggest that long‐term monitoring should cover both currently suitable areas and their higher‐latitude margins. Maintaining freshwater and estuarine environmental quality may help preserve conditions relevant to different life‐history stages (Zhang et al. 2023). Future monitoring should also incorporate recruitment, abundance, and long‐term population dynamics to evaluate whether projected changes in environmental suitability correspond to observed population responses (Jarnevich et al. 2015; Wu et al. 2024).

Future research should integrate verified spawning and nursery records, standardized field surveys, river‐flow direction, catchment connectivity, physical barriers, shipping pathways, and demographic data (Barber‐O'Malley et al. 2022; Robinson et al. 2019). These data would help link predicted suitability to actual dispersal and population processes, improving future distribution assessments and regional management strategies (Barber‐O'Malley et al. 2022; Guisan and Thuiller 2005; Jarnevich et al. 2015).

## 5 Conclusions

This study integrated global and regional ensemble species distribution models, with separate terrestrial and nearshore components, to assess the current and future environmental suitability of *Eriocheir sinensis* . The regional models revealed both shared and region‐specific environmental associations. Terrestrial suitability was primarily associated with temperature, precipitation, and distance to the coastline, although the relative importance of these predictors varied among regions. Environmental conditions associated with high predicted suitability in Europe and North America extended towards lower annual mean temperatures and lower mean temperatures of the wettest quarter than those in the native range. By contrast, nearshore suitability showed more consistent associations with water depth, chlorophyll concentration, sea‐surface temperature, and salinity. Collectively, these findings demonstrate pronounced regional variation in the modeled environmental suitability of *E. sinensis* .

Future projections indicated an overall redistribution of suitable environmental conditions towards higher latitudes in the Northern Hemisphere, characterized by increasing suitability at higher latitudes and declining suitability along lower‐latitude margins rather than a uniform expansion of the potential suitable range. Terrestrial suitability exhibited more pronounced spatial changes than nearshore brackish‐water suitability, and the two model components did not fully correspond geographically in some regions, highlighting the importance of considering both environmental dimensions when assessing this catadromous species. The shared PCA‐env analysis further revealed contrasting regional trajectories: Europe showed relatively high retention of its current suitable environmental space; North America exhibited more pronounced contraction and redistribution under late high‐emission conditions; and the native range showed declining retention accompanied by redistribution towards higher latitudes within the predefined projection domain. Expansion into new environmental space remained limited across all regions, indicating that future changes were dominated by differences in the retention and reorganization of currently suitable environmental conditions. Overall, these results reveal region‐specific reorganization and contrasting responses of projected suitable environmental space under future climate change. MESS analysis further identified areas in which future projections involved environmental extrapolation, thereby highlighting locations requiring targeted validation. Integrating terrestrial, nearshore, and river‐associated spatial information provides a practical framework for identifying potential management priorities and refining regional monitoring and field surveys. Future integration of verified reproductive records, hydrological connectivity, dispersal information, and demographic data will help link predicted environmental suitability to observed species responses and improve assessments of future distributional change and invasion dynamics.

## Author Contributions

**He Gong:** data curation (lead), investigation (lead), methodology (lead), resources (equal), software (lead), writing – original draft (lead). **Xiaolong Yang:** funding acquisition (equal), methodology (equal), resources (equal), supervision (equal), writing – original draft (equal). **Gorka Bidegain:** methodology (equal), writing – review and editing (equal). **Yihang Wang:** resources (equal), validation (equal). **Yuqing Jiang:** methodology (equal), software (equal). **Tao Han:** funding acquisition (equal). **Jianyu Dong:** methodology (equal), supervision (equal). **Xiumei Zhang:** funding acquisition (equal), methodology (equal), supervision (lead).

## Funding

This work was supported by Central University Basic Research Fund of China (2024J001‐3), National Natural Science Foundation of China (41906115), and National Key Research and Development Program of China (2023YFD2401901).

## Conflicts of Interest

The authors declare no conflicts of interest.

## Supporting information


**Data S1:** Dataset.

**Table S1:** Environmental variables.
**Figure S1:** Pairwise correlations among environmental predictors retained in each model.
**Figure S2:** Optimization results of the MaxEnt model (A–C for Land Datasets, D–F for Marine Datasets).
**Figure S3:** Spatial block cross‐validation performance of the four SDMs. Points show the mean ROC‐AUC and TSS values, and error bars represent ± SD across spatial validation folds. EUR‐SDM, European model; G‐SDM, global model; N‐SDM, native‐range model; NA‐SDM, North American model.

## Data Availability

The environmental data used in this study are publicly available from the WorldClim and Bio‐ORACLE databases. Species occurrence records were compiled from field surveys, published literature, and GBIF database. The integrated occurrence dataset used in this study is provided as Supporting Information with this manuscript.
