# Supplementary material for: Assessing the Potential Distribution of Eriocheir sinensis Under Climate Change: Regional Contrasts and Spatial Mismatches in Terrestrial and Nearshore Suitability
Source: Ecol Evol. 2026 Oct 16;16(10):e74351. doi: 10.1002/ece3.74351 (PMC13675134; doi:10.1002/ece3.74351)
Supplement: Supplementary file 1 [file ECE3-16-e74351-s001.docx]

***Ecology and Evolution***

Supplementary Material to

**Assessing the potential distribution of Eriocheir sinensis under climate change: regional contrasts and spatial mismatches in terrestrial and nearshore suitability**

He Gong^a^, Xiaolong Yang^a*^, Gorka Bidegain^b,c^, Yihang Wang^a^, Yuqing Jiang^a^, Tao Han^a^, Jianyu Dong^d^, ^a^, Xiumei Zhang^a^**

^a^ *Fishery College, Zhejiang Ocean University, Zhoushan 316022, China*

^b^ *Department of Applied Mathematics, Facuty of Engineering Gipukkoq, University of the Basque Country (UPV/EHU), Plaza Europa 1, 20018, San sebastian, spain*

*^c^ Research Center for Experimental Marine Biology and Biotechnology, Plentzia Marine Station, University of the Basque Country (PiE-UPV/EHU), Areatza Pasealekua,48620, Plentzia, Spain*

^d^ *Fisheries College, Guangdong Ocean University, Zhanjiang, 524088, China*

* Corresponding author at: Fishery College, Zhejiang Ocean University, No. 1, Haida South Road, Changzhi Island, Zhoushan, China. *E-mail address:* [2022090@zjou.edu.cn (X. Yang);](mailto:xlyang198805@163.com;) [xmzhang1227@163.com](mailto:xmzhang1227@163.com) (X. Zhang)

Table S1. Environmental Variables

| Land-environment variable | Marine-environment variable |
| --- | --- |
|  |  |
| Bio1 Annual Mean Temperature | M-Bio1 Maximum Air Temperature |
| Bio2Mean Diurnal Range | M-Bio2 Mean Air Temperature |
| Bio3Mean Diurnal Range | M-Bio3 Minimum Air Temperature |
| Bio4 Temperature Seasonality | M-Bio4 Water Depth |
| Bio5Mean Diurnal Range | M-Bio5 Maximum Chlorophyll |
| Bio6Min Temperature of Coldest Month | M-Bio6 Mean Dissolved Oxygen |
| Bio7Temperature Annual Range | M-Bio7 Mean Nitrate |
| Bio8Mean Temperature of Wettest Quarter | M-Bio8 pH |
| Bio9 Mean Temperature of Driest Quarter | M-Bio9 Mean Phosphate |
| Bio 10Mean Temperature of Warmest Quarter | M-Bio10 Mean Salinity |
| Bio 11Mean Temperature of Coldest Quarter | M-Bio11 Mean Silicate |
| Bio12 Annual Precipitation | M-Bio12 Surface Sea Temperature Maximum |
| Bio13 Precipitation of Wettest Month | M-Bio13 Surface Sea Temperature Mean |
| Bio14 Precipitation of Driest Month | M-Bio14 Surface Sea Temperature Minimum |
| Bio15 Precipitation Seasonality | M-Bio15 Mean Chlorophyll |
| Bio16 Precipitation of Wettest Quarter |  |
| Bio17 Precipitation of Driest Quarter |  |
| Bio18 Precipitation of Warmest Quarter |  |
| Bio19 Precipitation of Coldest Quarter |  |
| Bio20 Distance to Coast |  |

**G-SDM land**

**G-SDM Marine**

**Land**

**Land**

**Land**

Fig. S1. Pairwise correlations among environmental predictors retained in each model.

## MaxEnt model optimization and result

The SDMs for *E. sinensis* were developed utilizing the "Biomod2 (4.2.3)" package in R. Prior to model training, the ENMeval package was used to optimize the parameter combinations for the regularization frequency multiplier (RM) and feature combinations (FC) specific to the MaxEnt model (Muscarella et al. 2014, Liu et al. 2020, Kass et al. 2021). The RM values ranged from 0.5 to 4, with increments of 0.5, while the FC types included H, L, LQ, LQH, LQHP, and LQHPT (Kass et al. 2021, Zhao et al. 2021). Model performance for each parameter combination was assessed using the Akaike minimum information criterion (AICc), the average difference in area under the receiver operating characteristic curve (AUC) between the training and test data sets (avg.diff.AUC), and the average 10% omission rate (avg.test.or10pct) (Phillips et al. 2017, Zhao et al. 2021).

Among the tested parameter combinations, the MaxEnt model applied to the global dataset achieved optimal performance for RM = 0.5 and FC = LQHPT, with an AICc of 0 and the lowest avg.diff.AUC. For the N-SDM data set, RM = 1 and FC = LQHPT achieved the highest performance, with an AICc of 0 and the lowest avg.diff.AUC. In both cases, the corresponding avg.test.or10pct values remained within a reasonable and relatively low range. These parameter combinations were therefore selected for MaxEnt modeling (Supplementary Information, Fig S2).

C

F

E

D

A

B

Fig. S2. Optimization Results of the MaxEnt Model (A, B, C for Land Datasets, D, E, F for Marine Datasets).

Fig S3. Spatial block cross-validation performance of the four SDMs. Points show the mean ROC-AUC and TSS values, and error bars represent ± SD across spatial validation folds. N-SDM, native-range model; EUR-SDM, European model; NA-SDM, North American model; G-SDM, global model.

# References

Kass, J. M., R. Muscarella, P. J. Galante, et al. (2021). "ENMeval 2.0: Redesigned for customizable and reproducible modeling of species’ niches and distributions." Methods in ecology and evolution 12(9): 1602-1608.

Liu, T., J. Wang, X. Hu, et al. (2020). "Land-use change drives present and future distributions of Fall armyworm, *Spodoptera frugiperda* (JE Smith)(Lepidoptera: Noctuidae)." Science of the Total Environment 706: 135872.

Muscarella, R., P. J. Galante, M. Soley‐Guardia, et al. (2014). "ENM eval: An R package for conducting spatially independent evaluations and estimating optimal model complexity for Maxent ecological niche models." Methods in ecology and evolution 5(11): 1198-1205.

Phillips, S. J., R. P. Anderson, M. Dudík, et al. (2017). "Opening the black box: An open‐source release of Maxent." Ecography 40(7): 887-893.

Zhao, G. H., X. Y. Cui, J. J. Sun, et al. (2021). "Analysis of the distribution pattern of Chinese Ziziphus jujuba under climate change based on optimized biomod2 and MaxEnt models." Ecological Indicators 132.
